# Whole genome sequencing reveals rare off‐target mutations and considerable inherent genetic or/and somaclonal variations in CRISPR/Cas9‐edited cotton plants

**DOI:** 10.1111/pbi.13020

**Published:** 2018-10-30

**Authors:** Jianying Li, Hakim Manghwar, Lin Sun, Pengcheng Wang, Guanying Wang, Hanyan Sheng, Jie Zhang, Hao Liu, Lei Qin, Hangping Rui, Bo Li, Keith Lindsey, Henry Daniell, Shuangxia Jin, Xianlong Zhang

**Affiliations:** ^1^ National Key Laboratory of Crop Genetic Improvement Huazhong Agricultural University Wuhan Hubei China; ^2^ Agricultural Bioinformatics Key Laboratory of Hubei Province College of Informatics Huazhong Agricultural University Wuhan Hubei China; ^3^ Department of Biosciences Durham University Durham UK; ^4^ Department of Biochemistry School of Dental Medicine University of Pennsylvania Philadelphia PA USA

**Keywords:** cotton, CRISPR/Cas9, off‐target, whole genome sequencing, somaclonal variation, pre‐existing/inherent variation

## Abstract

The CRISPR/Cas9 system has been extensively applied for crop improvement. However, our understanding of Cas9 specificity is very limited in Cas9‐edited plants. To identify on‐ and off‐target mutation in an edited crop, we described whole genome sequencing (WGS) of 14 Cas9‐edited cotton plants targeted to three genes, and three negative (Ne) control and three wild‐type (WT) plants. In total, 4188–6404 unique single‐nucleotide polymorphisms (SNPs) and 312–745 insertions/deletions (indels) were detected in 14 Cas9‐edited plants compared to WT, negative and cotton reference genome sequences. Since the majority of these variations lack a protospacer‐adjacent motif (PAM), we demonstrated that the most variations following Cas9‐edited are due either to somaclonal variation or/and pre‐existing/inherent variation from maternal plants, but not off‐target effects. Of a total of 4413 potential off‐target sites (allowing ≤5 mismatches within the 20‐bp sgRNA and 3‐bp PAM sequences), the WGS data revealed that only four are *bona fide* off‐target indel mutations, validated by Sanger sequencing. Moreover, inherent genetic variation of WT can generate novel off‐target sites and destroy PAMs, which suggested great care should be taken to design sgRNA for the minimizing of off‐target effect. These findings suggested that CRISPR/Cas9 system is highly specific for cotton plants.

## Introduction

The CRISPR (clustered regularly interspaced short palindromic repeat)‐associated protein 9 (Cas9) is a bacteria immune system that has facilitated for genome editing in plant biotechnology and functional genomics research (Jiang *et al*., 2013; Lawrenson *et al*., 2015; Li *et al*., 2015; Liang *et al*., 2014; Mao *et al*., 2013; Miao *et al*., 2013; Wang *et al*., 2018). The *Stretococcus pyogenes* Cas9 (*SpCas9*) use ~20‐nt of a single guide RNA (sgRNA) to recognize with canonical NGG and non‐canonical NAG or NGA protospacer‐adjacent motif (PAM) sequence (Jinek *et al*., 2012). Once the CRISPR/Cas9 system has been introduced into the cell, it will induce the DNA double strand breaks (DSBs) and result in on‐ and off‐target mutations through non‐homologous end joining (NHEJ). The Cas9 protein can bind and cleave genomic sites that are homologous to sgRNA, which may result in unwanted off‐target mutations (Jinek *et al*., 2012). For diploid plant species, such as *Arabidopsis* and rice, the mutation profile is relatively simple, with four potential editing types: homozygous mutations, biallelic mutations, heterozygous mutations and no mutation (Feng *et al*., 2014). However, polyploid species such as cotton potentially have more complex mutation profiles (Wang *et al*., 2018). Off‐target mutations in CRISPR/Cas9‐edited cells can further add complexity to the mutation analysis, and this is emerging as the major concern for this promising technology.

In the early stages of Cas9 research, unusually high frequencies of off‐target mutations were detected in human cancer cell lines (Fu *et al*., 2013). The highly sensitive genome‐wide methods have been developed and applied in the detection of off‐target mutations in animal and human cell lines (Cameron *et al*., 2017; Hu *et al*., 2016; Kim *et al*., 2015; Kuscu *et al*., 2014; Ran *et al*., 2015; Tsai *et al*., 2015, 2017). With determination of the crystal structure of Cas9 and optimization of guide RNA design, off‐target mutations have been significantly decreased (Jinek *et al*., 2014). Recent reports show a moderate off‐target mutation frequency and this frequency is closely linked to the sgRNA's specificity. The off‐target number ranges from 0 to up to 150 mutations per genome in human cell lines (Frock *et al*., 2015). All these methods have been developed for the off‐target detection in animal or human cells, and then seldom have been successfully applied to study plant genome editing.

Actually, the off‐target effect is rare in plants. Several previous reports claimed that there is no mutation of potential off‐target sites in Cas9‐edited *Arabidopsis* (Feng *et al*., 2014), rice (Zhang *et al*., 2014) and tomato (Nekrasov *et al*., 2017). However, these studies exhibited several defects: (i) The number of the sequencing samples is low and absent the effective control; (ii) Moreover, these reports did not investigate the genetic variation of the maternal plants used for genetic transformation; and (iii) the detected potential off‐target sites were limited. Therefore, it is quite necessary to detect off‐target mutation at a genome‐wide level. Recently, a highly sensitive screen for genome‐wide CRISPR/Cas9 nuclease off‐target effect was reported in maize genome editing using CIRCLE‐seq method (Lee *et al*., 2018). A very meaningful off‐target analysis was conducted in Cas9‐ and Cpf1‐edited rice through whole genome sequencing (Tang *et al*., 2018). The authors analyzed the control with multiple background and nuclease edited rice suggested that off‐target mutations caused by Cas9 and Cpf1 nucleases are negligible when compared to somaclonal variations and spontaneous mutations.

Compared to these diploid species, cotton (*Gossypium hirsutum*) contains a more complex genetic structure and larger genome. Cotton is an allotetraploid (2*n* = 4*x* = 52, AADD) with a genome size of 2.5 Gb (Zhang *et al*., 2015). Moreover, *G. hirsutum* has homologous residings on each At‐ and Dt‐subgenome, suggesting that four alleles for each homologous pair of genes in the cotton genome. Allotetraploidy of cotton potentially will significantly complicate the mutation profile as well as the off‐target effects in CRISPR/Cas9‐edited plants. During the long‐term tissue culture process, some genetic variability is known as somaclonal variation, and can lead to genetic change at high frequency (Jin *et al*., 2008). These variations are induced by its long‐time maintenance under artificial conditions, leading to disorganized cell division *in vitro* and by the exposure of propagated tissue to high concentrations of plant growth regulators (Jiang *et al*., 2011). We therefore aimed to distinguish between potential off‐target mutations of CRISPR/Cas9 and somaclonal variation or/and pre‐existing/inherent variation from maternal plants in regenerated transgenic crop plants.

Here, we report results from a large‐scale whole genome sequencing (WGS) CRISPR/Cas9 library in three target genes. The WGS results indicated that the pre‐existing/inherent genetic or/and somaclonal variations during plant tissue culture process is more than the CRISPR off‐target effects. We demonstrated that the CRISPR/Cas9 system did not cause larger number of off‐target mutations in cotton plants. Furthermore, pre‐existing/inherent genetic variation of transgenic maternal plants can generate novel off‐targets. These findings also provided insights into great care should be taken when selecting a suitable reference genome to design sgRNA.

## Results

### Whole genome sequencing of CRISPR/Cas9‐edited cotton plants, WT and negative plants

**Figure 1:**
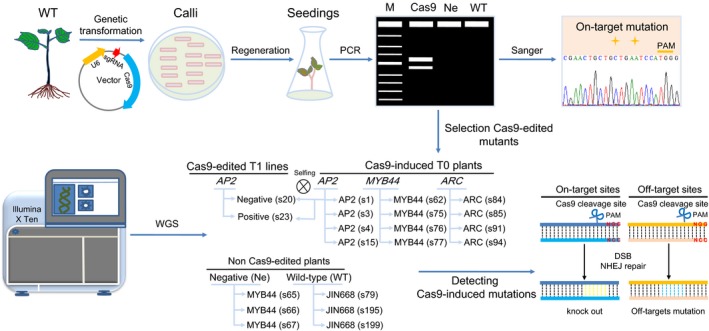
The whole genome sequencing analysis for the on‐ and off‐target mutations in CRISPR/Cas9‐edited cotton plants. Schematic diagram of the whole procedure of CRISPR/Cas9 system for gene editing in transgenic cotton plants. Whole genome sequencing was applied to 12 plants from Cas9‐edited T0 generation, targeting three endogenous genes (*AP2*,*MYB44* and *ARC*, four T0 plants from each target gene), two plants from T1 generation, three negative plants (Ne) in T0 generation and three wild‐type (WT) control cotton plants to detect the on‐ and off‐target mutations. As for the two T1 plants, they are derived from the same transgenic *AP2* T0 plant. Cas9‐negative plants contained the edited target site without the T‐DNA (no CRISPR/Cas9 fragment). Cas9‐positive plants contained the edited target site as well as the T‐DNA (with CRISPR/Cas9 fragment). The number in the brackets e.g. AP2 (s1) represents the plant or line number used for the WGS.

Recently, we established the CRISPR/Cas9 (*Streptococcus pyogenes* Cas9) system in cotton with high efficiency. Interestingly, no off‐target mutations were detected by targeted deep sequencing in the top 26 potential off‐target sites of four sgRNAs, suggesting that the CRISPR/Cas9 system is an efficient and reliable tool for allotetraploid cotton genome editing (Wang *et al*., 2018). To further investigate the specificity of CRISPR/Cas9 system on the genome‐wide scale in cotton genome editing, three target genes – encoding the AP2/B3‐like transcription factor (*AP2*), *MYB44* transcription factor (*MYB44*) and nucleotide‐binding (NB)‐ARC domain‐containing disease resistance protein (*ARC*) were edited using CRISPR/Cas9 in this study (Li *et al*., 2016). We performed whole genome sequencing in T0 generation plants and T1 lines with an approximate 35× sequencing depth (Table S1). To distinguish between Cas9‐edited mutations, somaclonal variations due to the tissue culture process and pre‐existing/inherent variations, three wild‐type (WT, *G. hirsutum* cultivar JIN668), three independent T0 negative (Ne) transgenic cotton plants undergoing tissue culture and plant generation but without the T‐DNA insertion (no CRISPR/Cas9 component), 12 independent T0 Cas9‐edited plants, and two T1 lines from *AP2* T0 parent were sequenced (Figure 1). For each gene, two sgRNAs were designed and sgRNAs target sites are shown in Table S2. Therefore, six sgRNAs were investigated for on‐target and off‐target mutations analysis.

### Detection of on‐target mutations at the genome‐wide scale

**Figure 2:**
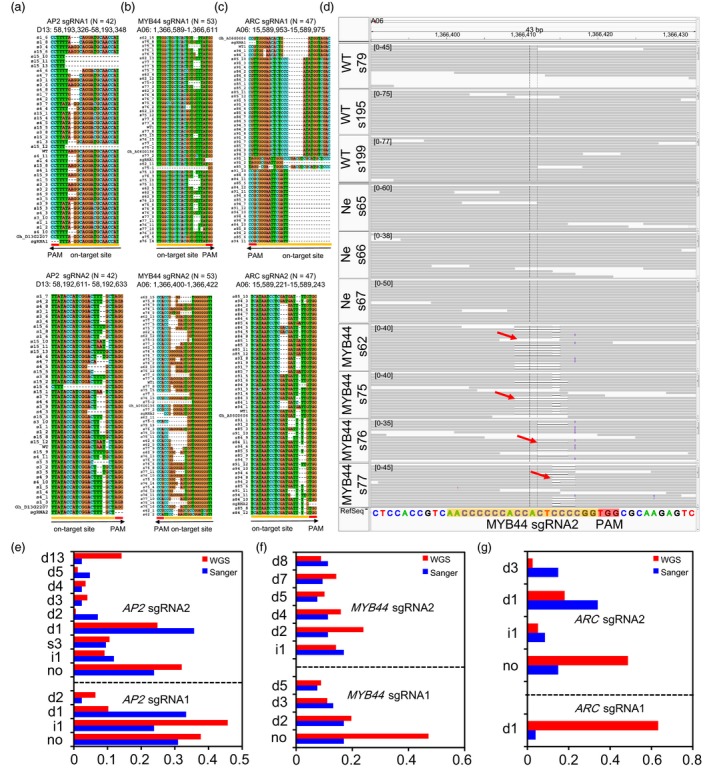
Whole genome sequencing and Sanger sequencing confirm on‐target mutations. (a–c) Sanger sequencing validate six sgRNA mutations of three endogenous genes. The orange and red lines represent the sgRNA and PAM sequences, respectively. The labelling on the side of the sequence alignment represent the each clones, and on top label represent on‐target genome coordinate, respectively. Black arrows indicate sgRNAs direction. (d) Whole genome sequencing analysis at sgRNA2 target region of *MYB44* in three wild‐type, three negative plants and four Cas9‐edited *MYB44* plants by Integrative Genomics Viewer (IGV). The number in the square brackets e.g. [0–45] represent the WGS supporting sequence reads with on‐target sites and the pileup strip represent Cas9‐edited *MYB44* plants a heterozygous deletion of 43‐bp in exon 1 of *MYB44*. Red arrows indicate DNA cleavage sites in different Cas9‐edited cotton plants. Orange and red box represent sgRNA sequence and PAM sequence, respectively. The sequences alignment of other five sgRNAs region in the three endogenous target genes are showed in Figures S2–S4. (e–g) The comparison of WGS and Sanger sequencing mutation frequencies for on‐target site. The ‘d’, ‘i’, ‘s’ represent the deletion, insertion and SNP genotypes, respectively. The ‘no’ indicate no editing and other mutation types at the on‐target site.

First, we tested on‐target site mutations in six sgRNAs of CRISPR/Cas9‐edited lines by Sanger sequencing (Figure 2a–c). The result showed that the efficiency of gene editing at each on‐target site was 70.1%–100%. Multiple types of mutations were detected at the specific on‐target sites (Figure S1a and Table S3). This editing efficiency is comparable to the efficiency (66%–100%) when editing exogenous transgene *Discosoma red fluorescent protein* (*dsRed2*) and an endogenous cotton gene *GhCLA1* in our recent report (Wang *et al*., 2018). We found no bias on the editing efficiency between At‐ and Dt‐subgenome loci based on WGS data, but different mutation patterns (Figure S1b). Further, analysis of the WGS data clearly reveals specific on‐target mutations in the CRISPR/Cas9‐edited transgenic cotton plants but not in WT, nor in negative control plants (Figures 2d and S2–S4). We detected 11 mutation types in the two sgRNAs of *AP2*, nine mutation types in the two sgRNAs sites of *MYB44* and four mutation types in the two *ARC* sgRNAs between WGS and Sanger sequencing. The WGS and Sanger sequencing data revealed that most of Cas9‐generated mutations are deletions (Figure 2e–g). We found a positive linear correlation (Pearson's correlation coefficient, *R* ^2^ = 0.69, *P *<* *0.001) between WGS and Sanger sequencing for the two sgRNAs mutation frequencies of the *AP2* gene (Figure S5). These results confirmed that six on‐target sites exhibited multiple mutation types and different mutation frequencies from three target genes.

### Inherent genetic variation or/and somaclonal variation in Cas9‐edited plants, WT and negative plants

**Figure 3:**
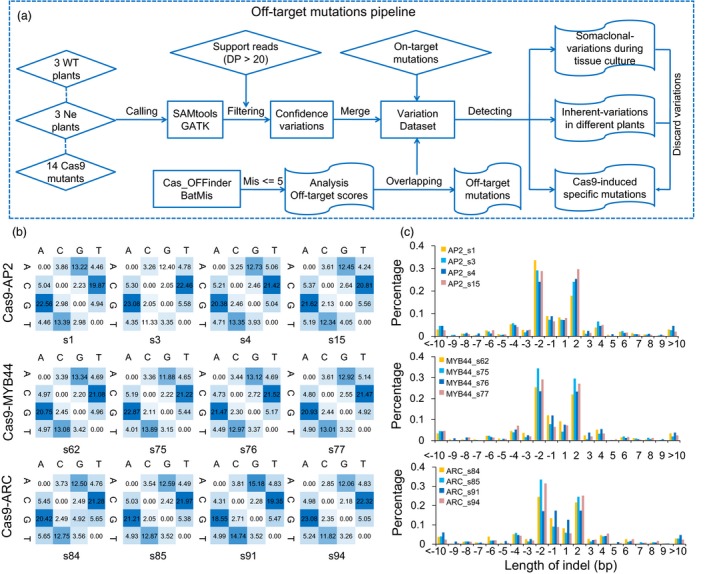
Genome‐wide analysis of variations in Cas9‐edited cotton plants during tissue culture process. (a) The bioinformatics pipeline for the off‐target mutations analysis. (b) Heatmap represents the percentage of specific mutation type in Cas9‐edited transgenic cotton plants. (c) Length of indels in different Cas9‐edited plants.

To evaluate the potential off‐target mutations of CRISPR/Cas9 in cotton plants, two standard computational methods were applied to detect all the variations, including SNPs and indels in the edited plants, negative and WT control plants compared with the cotton reference genome (Zhang *et al*., 2015; Figure 3a). Compared to the reference genome (*G. hirsutum* cultivar: TM‐1), the number of SNPs and indels in three WT plants (JIN668) are approximately 1 210 509–1 277 072 and 135 845–152 535, respectively. There are in a similar range to the genetic variation seen among different cotton cultivars (Wang *et al*., 2017; Table S4). To investigate pre‐existing mutations in the three WT cotton plants, we identified 28 054 SNPs and four indels with high confidence. The data suggested that the pre‐existing mutation ratio is ~2.8 × 10^−6^, which is much higher than in rice (Tang *et al*., 2018). However, there is no significant difference of variation number when comparing to Cas9‐edited lines and negative lines, whereas there is considerable genetic variation among different individual WT plants (Table 1). Analysis of the variation in Cas9‐edited and negative plants compared with WT plants as well as cotton TM‐1 reference genome found that the average SNP number was 65 152 and indel number was 14 415 (Table 1). An average 34.68% SNPs and 50.57% indels in negative control plants were found to overlap with variations in Cas9 (+) plants, suggesting that these variations might be caused by somaclonal variation during tissue culture, or/and inherent variations and the mutations induced by Cas9 endonuclease (Table 1). For example, several variations in the *AP2*,* MYB44*,* ARC* transgenic plants were observed due to somaclonal variation or/and inherent variation. These variations located in the regions without the homology with the sgRNAs (Figure S6). The 15 210–32 412 SNPs and 6316–8040 indels present in Cas9‐edited cotton plants, but not in WT, nor in negative control plants (Table 1). After filtering the shared variation, 4188–6404 SNPs and 312–745 indels were detected in *AP2‐*,* MYB44‐*,* ARC*‐edited plants and all these variations were only present in the Cas9‐edited cotton plants (Table 1). We found a small number of sample‐specific variations among Cas9 transgenic cotton plants (Figure S7). When these variations were annotated against the cotton reference genome, most variations occurs in intergenic region (Student's *t*‐test, *P *<* *2.86e‐11; Table S5). For the SNP mutation types, a slight preponderance of transitions over transversions was detected (Chi‐square test, *P *<* *0.001), in which C to T (19.35%–22.46%) and G to A (18.55%–23.08%) were the most two frequent mutations in independent Cas9‐edited plants (Figure 3b). The most abundant indels were 1~2‐bp in length (Figure 3c). Surprisingly, the flanking 20‐bp regions of these variation sites did not show any homology with the six sgRNAs.

**Table 1:** The summary of total variations in wild‐type, negative and CRISPR/Cas9‐edited cotton plants

Description	Lines vs Ref		Plants vs Ref/WT		Plants vs Ref/WT/Ne		Private variations	
Plants	SNP	Indel	SNP	Indel	SNP	Indel	SNP	Indel
WT (s79)	1 211 622	149 327	–	–	–	–	–	–
WT (s195)	1 214 683	152 535	–	–	–	–	–	–
WT (s199)	1 210 509	148 567	–	–	–	–	–	–
Negative (s65)	1 203 206	149 636	69 242	13 080	–	–	–	–
Negative (s66)	1 217 124	148 842	62 882	13 431	–	–	–	–
Negative (s67)	1 209 155	135 845	82 264	19 840	–	–	–	–
Cas9‐*AP2* (s1)	1 266 777	150 156	61 648	13 870	15 210	6935	4188	500
Cas9‐*AP2* (s3)	1 284 258	150 421	68 483	14 940	18 540	7736	4893	527
Cas9‐*AP2* (s4)	1 270 814	150 405	67 517	13 691	19 415	6707	5976	549
Cas9‐*AP2* (s15)	1 260 704	149 367	61 976	14 292	16 704	7282	4345	495
Cas9‐*AP2* (s20)	1 262 125	149 979	62 580	14 224	32 591	7950	6096	777
Cas9‐*AP2* (s23)	1 258 810	149 834	66 866	14 204	32 412	8040	5984	859
Cas9‐*MYB44* (s62)	1 259 373	149 594	64 312	13 508	21 368	6596	6404	532
Cas9‐*MYB44* (s75)	1 268 973	150 803	59 756	14 353	17 179	7123	2807	312
Cas9‐*MYB44* (s76)	1 261 093	150 637	61 338	13 064	18 726	6316	4595	484
Cas9‐*MYB44* (s77)	1 261 356	150 166	61 323	13 599	18 845	6684	5604	660
Cas9‐*ARC* (s84)	1 269 877	151 413	66 408	14 202	25 447	6884	5839	593
Cas9‐*ARC* (s85)	1 277 072	149 570	65 577	15 603	25 114	7852	5397	745
Cas9‐*ARC* (s91)	1 245 699	145 824	64 627	14 312	26 249	7035	4405	378
Cas9‐*ARC* (s94)	1 264 623	149 644	60 793	14 837	22 497	7406	4660	578

The ‘lines vs Ref’ represent the variation of each plant compared to TM‐1 reference genome using SAMtools and GATK tools (Table S4). The ‘lines vs Ref/WT’ represent the variations of each plant aligned to wild‐type (WT) and TM‐1 reference genome. Similarly, the ‘lines vs Ref/WT/Ne’ represent the variation of each Cas9‐edited transgenic plants aligned to WT, negative plants. Sample‐specific variations in three WT plants have the same genotype as three negative plants, but differ from each Cas9‐edited plants. Sample‐specific variations (including tissue culture variations, or/and inherent variations or/and Cas9‐edited mutations) were annotated by ANNOVAR (Table S5).

### Low frequency off‐target mutations detected in Cas9‐edited plants

To detect all the potential off‐target mutations, six sgRNA and their PAM sequences were aligned with the TM‐1 reference genome using CRISPR‐P and Cas‐OFFinder software (Bae *et al*., 2014; Liu *et al*., 2017). With ≤5 mismatches in the sgRNA and PAM sequences, there were 3296 (PAM: NGG), 410 (PAM: NAG) and 707 (PAM: NGA) potential off‐target sites identified (Table S6, Figure S8 and Appendix S1). A very low off‐target mutation frequency (four indels, PAM: NGG) was detected in different CRISPR/Cas9 transgenic lines by WGS (Table 2). Interestingly, there was no mutation detected by WGS at the most likely 10 off‐target sites (Table S7), according to the scoring system of off‐target sites by a high‐throughput analysis in mammalian cells (Hsu *et al*., 2013).

**Table 2:** Identification of off‐target mutations in Cas9‐edited plants by whole genome sequencing

Cas9‐edited plants/sgRNA	Cas9 mutations/No. of NGG sites (Ratio%)	Cas9 mutations/No. of NAG sites (Ratio%)	Cas9 mutations/No. of NGA sites (Ratio%)
*AP2* (sgRNA1)	0/441 (0.00)	0/57 (0.00)	0/155 (0.00)
*AP2* (sgRNA2)	0/765 (0.00)	0/55 (0.00)	0/83 (0.00)
*MYB44* (sgRNA1)	0/683 (0.00)	0/55 (0.00)	0/151 (0.00)
*MYB44* (sgRNA2)	2/182 (1.10)	0/8 (0.00)	0/15 (0.00)
*ARC* (sgRNA1)	2/341 (0.59)	0/66 (0.00)	0/54 (0.00)
*ARC* (sgRNA2)	0/884 (0.00)	0/169 (0.00)	0/249 (0.00)

The six sgRNA sequences were aligned TM‐1 reference genome using Cas‐OFFinder and BatMis tools with up five mismatch, the 3296 (NGG), 410 (PAM: NAG) and 707 (PAM: NGA; Table S6 and Appendix S1).

**Figure 4:**
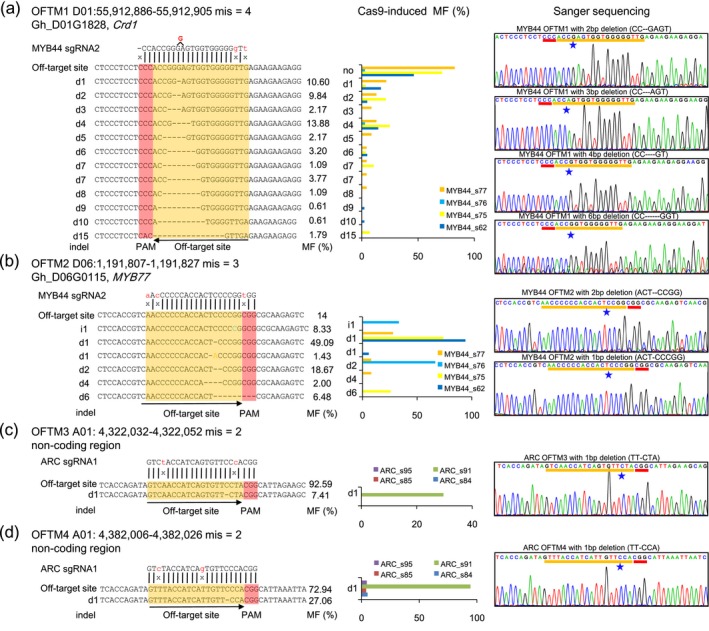
The identification of potential off‐target mutations in Cas9‐edited cotton plants by whole genome sequencing and Sanger sequencing. (a) Off‐target mutations in promoter region of the *MYB44* gene. (b) Off‐target mutations in exon region of the *MYB44* gene. (c,d) Off‐target mutations in non‐coding regions of *ARC* gene. The left panel showed the different off‐target mutation types. Mismatch (on‐target site vs off‐target site) nucleotides are showed in ‘*x*’, the PAM and off‐target region are showed in red and orange rectangular. The on top of left panel represent the each off‐target genome coordinate. Black arrows represent sgRNA transcription direction. The OFTM represent the off‐target site mutation in Cas9‐edited cotton plants. The Cas9‐edited MF represents the mutation frequency (MF) in different Cas9‐edited cotton plants compared to WT plants. The MF in the left panel showed the average mutation frequency per plant. The middle panel exhibited indel frequency in different Cas9‐edited plants. The right panel illustrated the Sanger sequencing data at off‐target sites. The stars in the right panel represent the cleavage sites.

Sanger sequencing was used to confirm these off‐target mutations with PAM (NGG) site. The Cas9‐edited *AP2* lines had no off‐target mutation detectable by WGS. In the *MYB44* Cas9‐edited plants, two off‐target sites were detected in the promoter region of dicarboxylate diiron protein (*Crd1*; Gh_D01G1828, OFTM1) and the first exon of *MYB77* (Gh_D06G0115, OFTM2; Figure S9). The WGS data revealed the length frequencies of deletion at *Crd1* and *MYB77* off‐target sites (Figure 4a,b, middle panel). The indels were 1~4‐bp in length at *Crd1* off‐target sites and 1‐bp at the *MYB77* off‐target sites. We also detected *ARC* off‐target mutations and revealed 1‐bp deletions in two off‐target sites (OFTM3/4; Figures 4c,d and S10). The Cas9‐generated 1‐bp deletion at two off‐target sites in the non‐coding region was also validated by Sanger sequencing in both Cas9‐edited and WT plants (Figure 4c,d). We also found the deletion frequencies varied in independent Cas9‐edited plants. These results indicated that the low frequency off‐target mutations were low in Cas9‐edited cotton plants, even there were some unpredicted mutations in the regions that were not homologous to sgRNA target sites.

### Genetic variation among cultivars can generate novel off‐target sites

As mentioned previously, the WT used for genetic transformation is *G. hirsutum* cultivar JIN668, which contains more than 1 million high‐confidence SNPs and indels compared to the cotton reference genome (Table 1). To investigate the new off‐target sites or PAMs created by these inherent genetic variations in the WT, we reanalyzed the aforementioned predicted 4413 potential off‐target sites from six sgRNAs. When comparing the genetic variation in maternal WT with the reference genome, more than 61 SNPs and indels were identified in the 4413 potential off‐target sites, which can alter on‐ and off‐target variations by increasing and decreasing the number of mismatches with sgRNAs as well as PAM sites. Therefore, we defined these off‐target sites with variations in the WT genomes as ‘Novel’ potential off‐targets. For example, one PAM (NGG) generated a novel site (NCG) at the target site of *AP2* gene (Table S8). Moreover, there were 39, 6 and 10 novel off‐target sites identified with NGG, NAG and NGA ‘PAM’ site, respectively, at six sgRNA target sites in the *AP2*,* MYB44* and *ARC* genes (Tables 3 and S8). These results demonstrated that there is considerable genetic variability in the maternal cultivar used for genetic transformation, which will affect the accuracy of sgRNAs if these sgRNAs are designed based on a different reference genome.

**Table 3:** Newly generated off‐target sites or PAMs by genetic variations in the WT plants

Target	*AP2*		*MYB44*		*ARC*	
sgRNA	sgRNA1	sgRNA2	sgRNA1	sgRNA2	sgRNA1	sgRNA2
Off‐target site (NGG)	4/441	7/765	12/683	4/182	4/341	8/884
PAM site (NGG)	0/441	3/765	1/683	0/182	0/341	1/884
Off‐target site (NAG)	0/57	2/55	0/55	1/8	0/66	3/169
PAM site (NAG)	0/57	0/55	0/55	0/8	0/66	0/169
Off‐target site (NGA)	2/155	2/83	3/151	0/15	0/54	3/249
PAM site (NGA)	0/155	0/83	0/151	0/15	1/54	0/249

The number in front of the ‘/’ represent the novel off‐target site in WT and number behind the ‘/’ represent the total potential off‐target sites in reference genome, respectively.

### Investigation of spontaneous mutations and the inheritance of Cas9‐edited mutations

To investigate and estimate the level of spontaneous mutations across generation, we analyzed the WGS data from T0 to T1 generation. For this purpose, three WT plants, three Negative plants, and 12 T0 Cas9‐edited plants were analyzed based on the WGS data to exclude Cas9‐induced mutations and pre‐existing mutations. After filtering the pre‐existing and Cas9‐induced mutations, 466 SNPs and 77 indels were identified as spontaneous mutations from T0 (s1) to T1 (s20, s23) in this experiment. The inherited mutation rate was ~5.43 × 10^−8^ per site per tetraploid genome from T0 to T1, which is consistent with previous report in rice with the spontaneous mutation rates (~5.4 × 10^−8^; Tang *et al*., 2018) and maize (2.17~3.87 × 10^−8^; Yang *et al*., 2017).

To test the inheritance of on‐target and off‐target mutations in the Cas9‐edited plants, transgenic *AP2* plants were evaluated at on‐ and off‐target sites from the T0 (s1) to T1 (s20, s23) generations. The result showed that the on‐target mutation in Cas9‐edited *AP2* T0 plant could be transmitted to T1 progeny at two sgRNAs loci (Table 4 and Figure S11). More importantly, there was no new editing detected at the target sites in T1 progenies. As expected, we did not detect any off‐target mutations (SNPs/indels) in either T0 and T1 generation from the WGS data, suggesting that the inheritance of Cas9‐edited mutation is very faithful between different cotton generations.

**Table 4:** The inheritance of Cas9‐edited mutations from T0 to T1 in AP2 Cas9‐edited plants

AP2 sgRNA1	Type	s1 (T0, Cas9+)		s20 (T1, Cas9−)		s23 (T1, Cas9+)	
		D13	D12	D13	D12	D13	D12
ATGGTTGCATCCTGCCTAAA**AGG**	no	0	0	0	0	0	0
ATGGTTGCATCCTGCCTTAAA**AGG**	i1	0	0	0	0	0	0
ATGGTTGCATCCTG‐‐TAAA**AGG**	d2	18	7	0	3	26	11
ATGGTTGCATCCTGC‐‐AAA**AGG**	d2	23	41	54	27	11	31
*AP2 sgRNA2*		D13	A12	D13	A12	D13	A12
**CCT**AGCAAAGTCCGATGGTATAA	no	24	22	32	29	0	27
**CCT**AGCAAAAGTCCGATGGTATAA	i1	0	0	2	3	0	0
**CCT**AGATTAGTCCGATGGTATAA	s3	0	0	0	0	0	0
**CCT**AGCA‐AGTCCGATGGTATAA	d1	1	13	1	0	21	0
**CCT**AGCA‐‐GTCCGATGGTATAA	d2	0	0	0	0	0	0
**CCT**AGC‐‐‐GTCCGATGGTATAA	d3	1	0	0	1	0	0

## Discussion

To increase Cas9 accuracy by protein engineering, marked improvements have been achieved as a result of decoding the crystal structure of Cas9, identifying Cas9 orthologs, identifying CRISPR variants and single/paired nickases (Tycko *et al*., 2016). Although these strategies allow engineering of the Cas9 protein, higher specificity also can be obtained by improving the sgRNAs computational tools to guide RNA prediction.

However, recent research from Schaefer *et al*. describes 117 small insertions and deletions (indels) and 1397 single‐nucleotide variations (SNVs) using whole genome sequencing in two Cas9‐treated mice, which were absent in the uncorrected control (Schaefer *et al*., 2017). Although several groups questioned this research both in the mouse sample number (only two CRISPR‐treated mice were used) and the some group concerns about the method for the off‐target analysis, the frequency of off‐target mutations following CRISPR/Cas9 gene editing are of current interest (Kim *et al*., 2018; Lareau *et al*., 2018; Wilson *et al*., 2018).

In this study, we adopted the most widely used SpCas9 for genome editing. Since it shows higher on‐target editing efficiency and undetectable off‐target effects in cotton genome editing. We demonstrated that the most variations in Cas9‐edited cotton plants were due either to somaclonal variation or/and pre‐existing/natural variation from maternal plants, but not off‐target effects, which is consistent with previous report in rice (Tang *et al*., 2018). To investigate pre‐existing mutations in the three WT plants, we get high confidence 28 054 SNPs and four indels, the pre‐existing mutations is ~2.8 × 10^−6^, but much higher that the pre‐existing mutation rate in rice (Tang *et al*., 2018). This is maybe due to the excellent rice reference genome, but in cotton the considerable pre‐existing variations from one of subgenome or heterozygous and genome assembling error. Through sequencing reasonable Cas9‐edited cotton plants, we detected four *bona fide* off‐target mutations. Based on the off‐target homology and score, we revealed that highly homologous sequences have the potential Cas9 cleavage sites in seed sequence of the protospacer (Figure S8b). These findings suggest that CRISPR/Cas9 system is quite reliable and specific in plants.

The sgRNA design may largely determine the likelihood of off‐target effect. We revealed that genetic variations from maternal plants can alter PAMs and increase the risk of off‐target effects. Recently, two groups demonstrated that human genetic variation can alter the landscape of on‐target and potential off‐target sites genome‐wide, by creating and breaking their canonical PAM sequence in the CRISPR/Cas9 system (Lessard *et al*., 2017; Scott and Zhang, 2017). The current standard procedure is to design sgRNAs against the standard reference genome of relevance (i.e. Col‐0 in *Arabidopsis*, Nipponbare in rice and TM‐1 in cotton). However, in many plant species, a high quality reference genome is not available and usually the cultivars/genotypes with reference genome may not be widely used for genetic transformation (e.g. JIN668 rather than TM‐1 of cotton, and Zhonghua 11 rather than Nipponbare of rice, are widely used for genetic transformation). Any variations in the particular genomes of plant cultivars, the animal cell line or tissue, or strain of bacteria can adversely affect sgRNA specificity. In this study, more than one million SNPs and indels were found in WT plants compared with the reference genome, which generated novel off‐target sites and dramatically affected sgRNA specificity. Therefore, an exquisite level of understanding of genotypic variation through WGS analysis becomes an important initial step in sgRNA design, to increase specificity for CRISPR/Cas9 applications.

Once a reference genome for cultivars/cell/lines/strains of interest is available, the next important step is to choose appropriate tool for sgRNA design and off‐target prediction. To date, more than 10 computational tools [including CRISPR Design Tool (Hsu *et al*., 2013), and Cas‐OFFinder (Bae *et al*., 2014)] have been developed, mainly for animal genome editing (Tycko *et al*., 2016). We used the CRISPR‐P web tool to design sgRNA (Liu *et al*., 2017), which is widely used for the guide RNA design and the off‐target prediction for plant species (Ma *et al*., 2015; Soyk *et al*., 2017; Zhang *et al*., 2017). Moreover, it supports the design of guide sequences for various Cas9 orthologs as well as various CRISPR‐Cas systems like Cpf1. The undetectable off‐target effect in our previous report (Wang *et al*., 2018) and lower off‐target mutations imply that the CRISPR‐P is reliable for sgRNA design in plant species. Taken together, when designing sgRNAs, great care should be taken to consider the genetic variation from cultivar with different genetic background to minimize off‐target effect.

Currently, several methods are available to detect the off‐target mutation caused by sequence‐specific nucleases (SSNs) including targeted sequencing, Digenome‐seq (Kim *et al*., 2015), GUIDE‐seq (Tsai *et al*., 2015), SITE‐seq (Cameron *et al*., 2017), BLESS‐seq (Ran *et al*., 2015), ChIP‐seq (Kuscu *et al*., 2014), LAM‐HTGTS and whole genome sequencing (Hu *et al*., 2016). All these methods have strengths and weaknesses. These methods can detect genome‐wide direct labeling of DSBs, but only detect the DSBs present at the time of labeling (Zischewski *et al*., 2017). Among these methods, the WGS is very convenient and effective for identifying not only small indels and SNPs but also structural variations, such as major deletions, rearrangements, inversions and duplications (Veres *et al*., 2014). Therefore, a relatively high sequencing depth (>50) is required, which means high cost. The major limitation of the method is that it is difficult to detect the low frequency mutations in low sequencing depth.

In summary, the WGS data revealed thousands of novel SNPs/indels in Cas9‐edited cotton plants compared to WT, negative and cotton reference genome sequence. However, when compared to the sgRNAs, we found out that the majority of these variations loci lacked PAM sequence. We only detected four off‐target mutations in Cas9‐edited plants from 4413 predicted off‐targets based on the WGS data and these mutations were validated by Sanger sequencing. Notably, 61 potential off‐target sites and PAM were generated by genetic variation in WT plants. Also, there is no evidence of off‐target mutations from T0 to T1 generation. We therefore suggested that most variations in Cas9‐edited plants is induced by somaclonal variation during the tissue culture process, pre‐existing variation across germline, or/and inheritance from the maternal plants.

## Experimental procedures

### Computational sgRNA design and selection

The sgRNAs were designed according to CRISPR‐P web tool (Liu *et al*., 2017). The *AP2*,* MYB44* and *ARC* target genes were selected for further analysis (Li *et al*., 2016). First, we found all possible sgRNA sequences in specific target gene with GC content (40%~60%) and on‐target score (score value > 0.6). For the off‐target effect, the number of off‐target site mismatches was <5 with on‐target site. Then, the off‐target scores were calculated based on the scoring system of off‐target sites from previous research (Hsu *et al*., 2013). The detail steps of optimal CRISPR sgRNAs were filtered, including the sgRNA genome position, on‐target score, off‐target score and the number of potential off‐targets. For each target gene, two sgRNAs were designed in the coding region, namely sgRNA1 and sgRNA2. For the polyploid plants, we need to consider the homologous gene pair of At‐ and Dt‐subgenome to increase the specificity of the sgRNA designing, i.e. sgRNA1 and sgRNA2 of *AP2*, and ARC have two target loci in At‐ and Dt‐subgenome, respectively. The sgRNA1 and sgRNA2 of *MYB44* have one target site in At‐subgenome.

### Vector construction

The CRISPR/Cas9 vector used in this report is modified from *pRGEB32‐GhU6.9* according to our recent report (Wang *et al*., 2018). These two sgRNAs were integrated in a single vector, which included the fragments containing tRNA‐sgRNA1 and gRNA‐tRNA‐sgRNA2 fusion using pGTR as template, namely PTG (Xie and Yang, 2013), and then these two fragments were fused together with an overlapping extension PCR. The PTG fragment was ligated to *pRGEB32‐GhU6.9‐NPT II* expression vector, which was transformed into *Agrobacterium tumefaciens strain EHA105* for stable cotton transformation.

### *Agrobacterium*‐mediated transformation in cotton

Elite cotton cultivar (*Gossypium hirsutum*: Jin668) was used as the transformation receptor as described in our previous protocol (Li *et al*., 2018). Putative transgenic T0 plants were obtained and screened by Polymerase Chain Reaction (PCR) analysis. All the positive and negative plants then were transferred to a greenhouse to generate T1 seeds.

### On‐target analysis of CRISPR/Cas9‐edited plants

DNA from T0 and T1 mutated plants were subjected to PCR for the positive identification of mutants induced through using Cas9 forward and reverse primers (Table S9). Selected positive Cas9‐edited plants were amplified via PCR and the PCR products were ligated to pGEMT‐Easy vector for TA cloning with T4 DNA ligase (Promega, Madison). These clones were used for Sanger sequencing to detect on‐target mutations caused by Cas9 nuclease. On‐target site mutations are showed through WGS data in Figures S2–S4 using Integrative Genomics Viewer (IGV) tools (Robinson *et al*., 2011). The sequences were aligned using ClustalW between Cas9‐edited and WT plants.

### Genomic DNA isolation and library construction for Whole Genome Sequencing

Genomic DNA was extracted from young leaves of the three WT plants, three negative plants, 14 Cas9‐edited lines using Plant Genomic DNA kit (TIANGEN, Cat. #DP305‐03). For each sample, at least 5 μg DNA was used to construct sequencing library according to Illumina TruSeq DNA Sample Prep Kit (San Diego, CA). Final libraries were sequenced on an Illumina HiSeq X Ten (Paired‐end 150 bp) with an average 35× sequencing depth at the Novogene company (Beijing, China).

### Bioinformatics analysis for variant calling

The reference allotetraploid cotton genome (*Gossypium hirsutum*, TM‐1) and its annotation were downloaded from CottonGen (https://www.cottongen.org/). Scaffolds with sequence lengths less than 2000‐bp were discarded from further analysis. First, the raw reads were filtered with Trimmomatic (Version 0.32, MINLEN:75; Bolger *et al*., 2014). Then, clean reads were mapped TM‐1 genome (Zhang *et al*., 2015) using BWA (Li and Durbin, 2009) with default parameters. The SNPs were called with SAMtools (Li *et al*., 2009) software and Genome Analysis Toolkit (GATK; Mckenna *et al*., 2010; ‐stand_call_conf 30.0). For indels, the BAM files were realigned using the GATK with (‐T RealignerTargetCreator, IndelRealigner) to get accurate indel. To obtain high‐quality SNPs and indels, common variations detected by GATK and SAMtools using GATK software merged the (‐T Selectvariations) at least depth 20× for each site was retained for further analysis. Finally, the VCF files were merged from different groups with VCFtools (Danecek *et al*., 2011). The SNPs and indels were filtered with parameter (QD < 20.0 || ReadPosRankSum < −8.0 || FS > 10.0 || QUAL < $MEANQUAL). The final private variations were annotated by ANNOVAR software (Wang *et al*., 2010).

### Genome‐wide prediction off‐target cleavage sites and detection their mutations

The six sgRNAs were aligned against TM‐1 reference genome using BatMis and Cas‐OFFinder algorithm with up five mismatched as described (Bae *et al*., 2014; Tennakoon *et al*., 2012). The BatMis program was employed to perform sgRNA alignment with whole reference genome sequences. The off‐target sites were divided into NGG, NAG, NGA and others according to PAM site type using custom Perl script. The detail protocol for analysis in Cas9‐induced off‐target variations, somaclonal variations and inherent variations were detected. The pipeline is showed in Figure 3a. First, the variations present in three WT plants, where three negative plants have same genotype but differs from Cas9‐edited plants. All Cas9 lines were separated for further analysis. This step can discard somaclonal variation due to the tissue culture process or/and plant inherent variations from three negative plants. Secondly, analysis of independent Cas9‐edited plants variations compared to WT plants to filter inherent variations and individual plant tissue culture variations. This allows the identification of unique variations in each Cas9‐edited plant, which were called private variations. Finally, the candidate off‐target sites flanking 20‐bp were used to search the corresponding SNP/indel variations of Cas9‐edited plants to generate a likely off‐target mutation. The potential off‐target mutations were visualized in WT, negative, and Cas9‐edited plants by IGV tool to confirm the Cas9 nuclease induced mutations.

### Analysis genetic variation of potential off‐targets and PAMs between WT and cotton reference genome

In total, the 4413 off‐target sites with canonical PAMs (PAM = NGG or NAG or NGA) were used to analyze the inherent genetic variation impacts Cas9 endonucleases specificity. The WT (s79, s195, s199) variations (SNPs and indels, showed in Table 1) calling was performed with SAMtools and GATK, which then overlapped with off‐target and PAM sequences with the BEDTools package (Quinlan and Hall, 2010).

## Conflict of interest

The authors have declared that no competing interests exist.

## Supporting information


**Figure S1** The compared genome editing efficiency in Cas9‐edited plants.
**Figure S2** Confirmation of the AP2 on‐target mutation in two sgRNAs from WT, negative and CRISPR/Cas9 plants based on the WGS data.
**Figure S3** Confirmation of the MYB44 on‐target mutation in two sgRNA loci from WT, negative and CRISPR/Cas9 plants based on the WGS data.
**Figure S4** Confirmation of the ARC on‐target mutation in two sgRNA loci from WT, negative and CRISPR/Cas9 plants based on the WGS data.
**Figure S5** Scatterplot of on‐target site correlation of sanger sequencing and Whole genome sequencing.
**Figure S6** The somaclonal variation or/and inherent genetic variation were detected in AP2, *MYB44*,* ARC* Cas9‐edited plants and WT by IGV.
**Figure S7** The common and private SNPs/indels in four Cas9‐edited lines of AP2, MYB44, *ARC*.
**Figure S8** Genome‐wide prediction off‐target sites.
**Figure S9** The potential off‐target mutations in *MYB44* Cas9‐edited cotton plants.
**Figure S10** The potential off‐target mutations in *ARC* Cas9‐edited cotton plants.
**Figure S11** Confirmation of the *AP2* mutations of inheritance from T0 to T1 plants in two sgRNA loci by WGS data.

**Table S1** The depth of whole genome sequencing data.
**Table S2** Summary of target genes in CRISPR/Cas9 editing.
**Table S3** The different on‐target site mutation frequency of *AP2*,* MYB44*,* ARC* in each target site by Sanger sequencing.
**Table S4** The summary of variants calling by the SAMtools and GATK.
**Table S5** Genomic distribution of private variations in Cas9‐edited plants.
**Table S6** Summary of the off‐target sites across six sgRNA of three target genes.
**Table S7** Identification of most off‐target site mutations in CRISPR/Cas9 edited plants.
**Table S8** Summary of new off‐targets and PAMs in WT plants.
**Table S9** Primers used for on‐targets and off‐targets.

**Appendix S1** The number of potential off‐target sites were identified by CRISPR‐P and Cas‐OFFinder software with allowing up five mismatch for six sgRNAs.

## Data Availability

The potential off‐target sites used for mutations analysis are available as Appendix S1. All sequencing data used for the off‐target mutations are available from the author. High‐throughput sequencing data have been deposited in the NCBI Sequence Read Archive (SRA) BioProject ID: PRJNA380842.
