# Supplementary material for: Whole genome sequencing reveals rare off‐target mutations and considerable inherent genetic or/and somaclonal variations in CRISPR/Cas9‐edited cotton plants
Source: Plant Biotechnol J. 2018 Oct 30;17(5):858–68. doi: 10.1111/pbi.13020 (PMC6587709; doi:10.1111/pbi.13020)
Supplement: Supplementary file 1 — Figure S1 The compared genome editing efficiency in Cas9‐edited plants. Figure S2 Confirmation of the AP2 on‐target mutation in two sgRNAs from WT, negative and CRISPR/Cas9 plants based on the WGS data. Figure S3 Confirmation of the MYB44 on‐target mutation in two sgRNA loci from WT, negative and CRISPR/Cas9 plants based on the WGS data. Figure S4 Confirmation of the ARC on‐target mutation in two sgRNA loci from WT, negative and CRISPR/Cas9 plants based on the WGS data. Figure S5 Scatterplot of on‐target site correlation of sanger sequencing and Whole genome sequencing. Figure S6 The somaclonal variation or/and inherent genetic variation were detected in AP2, MYB44, ARC Cas9‐edited plants and WT by IGV. Figure S7 The common and private SNPs/indels in four Cas9‐edited lines of AP2, MYB44, ARC. Figure S8 Genome‐wide prediction off‐target sites. Figure S9 The potential off‐target mutations in MYB44 Cas9‐edited cotton plants. Figure S10 The potential off‐target mutations in ARC Cas9‐edited cotton plants. Figure S11 Confirmation of the AP2 mutations of inheritance from T0 to T1 plants in two sgRNA loci by WGS data. [file PBI-17-858-s001.pdf]

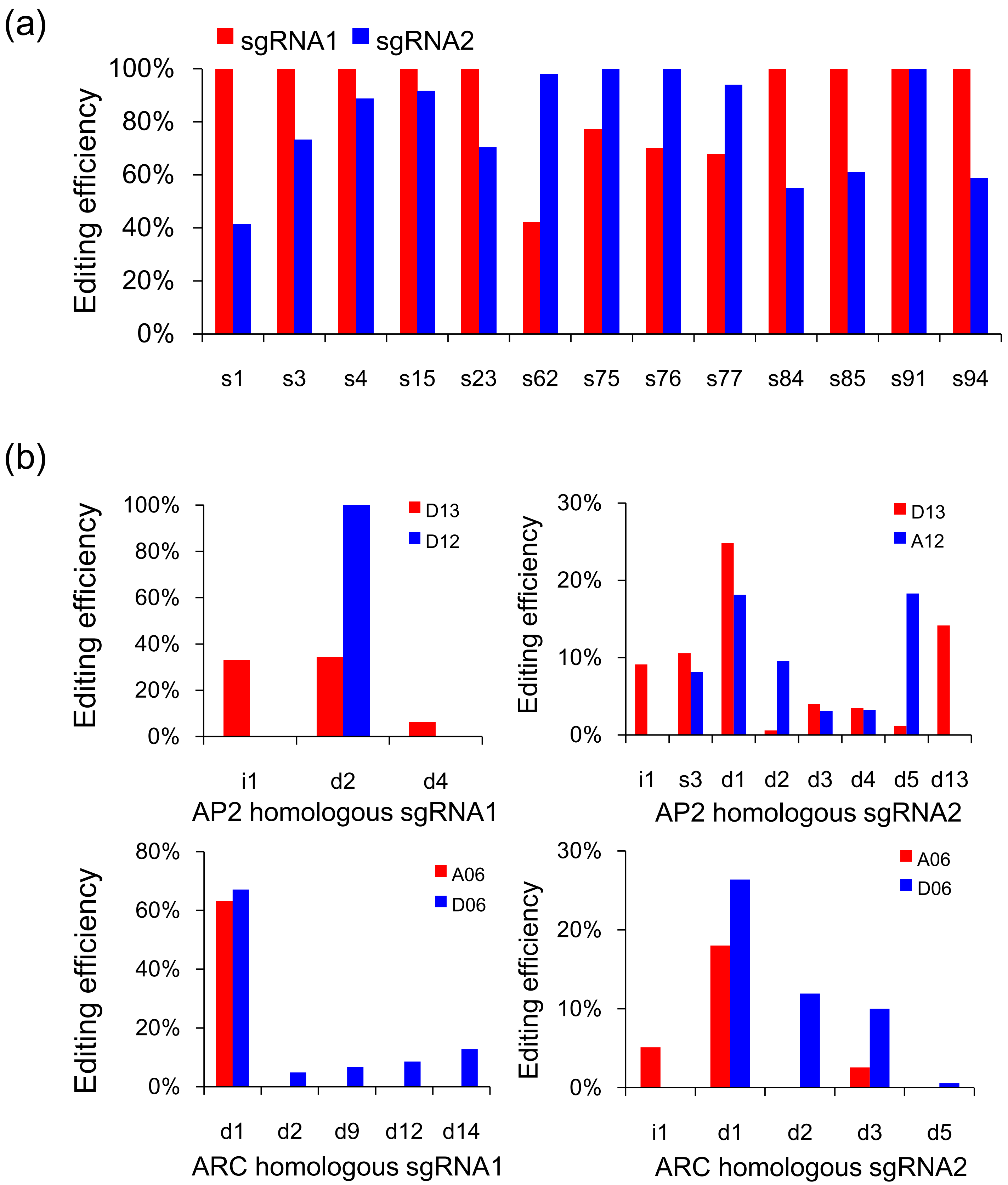

Figure S1 The compared genome editing efficiency in Cas9-edited plants. (a) The editing efficency in different Cas9-edited plants. (b) The compare genome editing efficiency of At and Dt-subgenome based on WGS data.

(a) AP2 D13 homologous sgRNA1

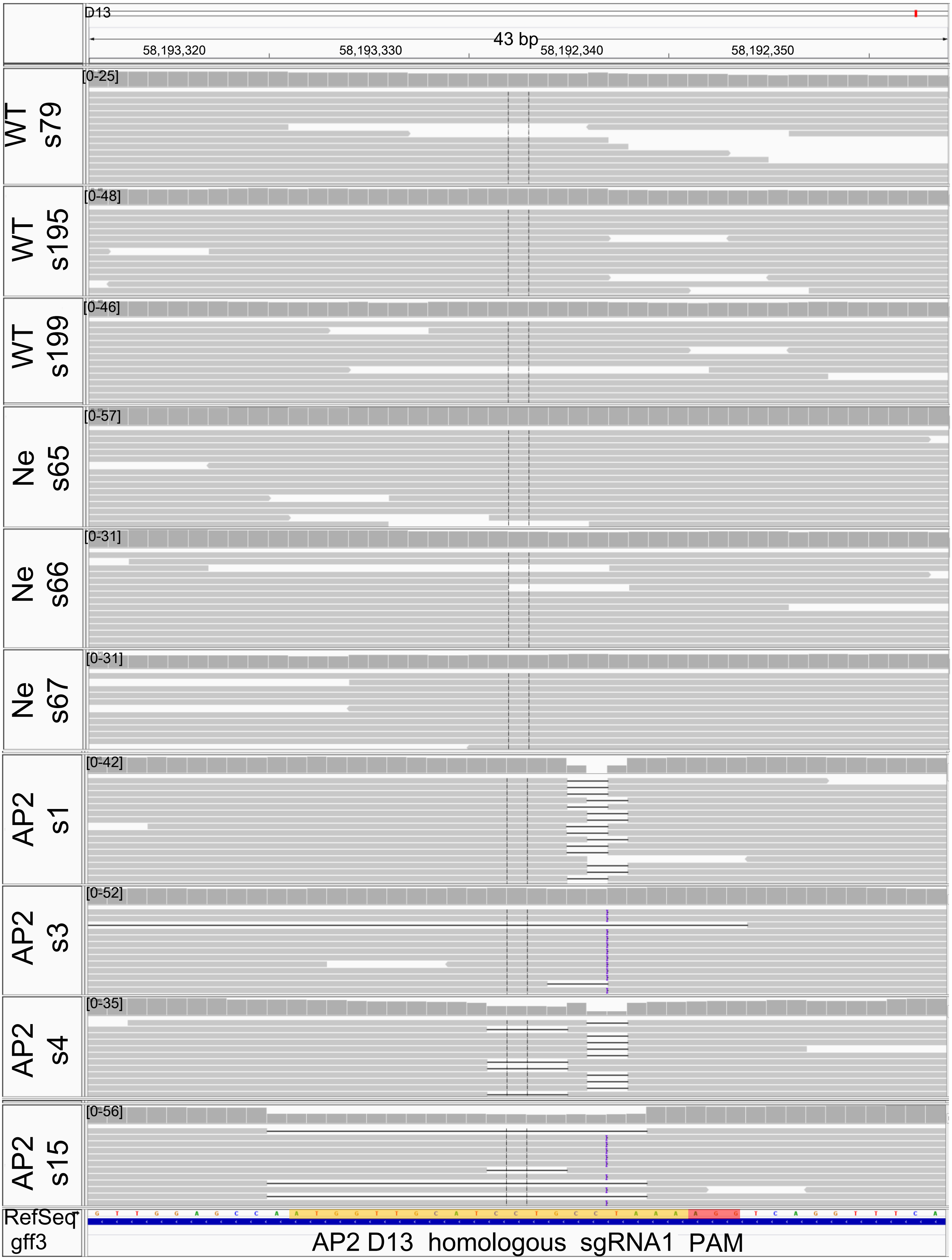

(b) AP2 D12 homologous sgRNA1

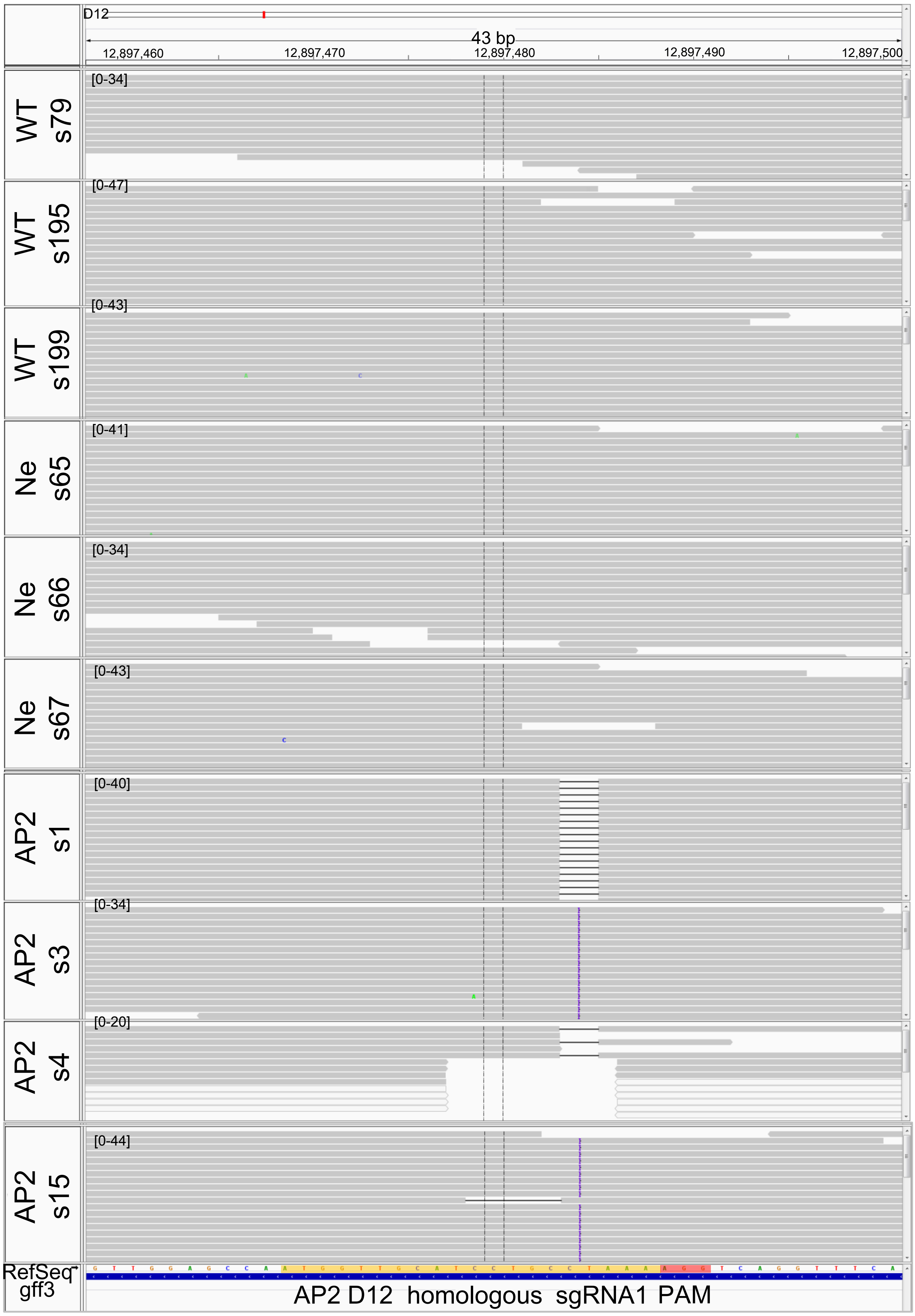

(c) AP2 D13 homologous sgRNA2

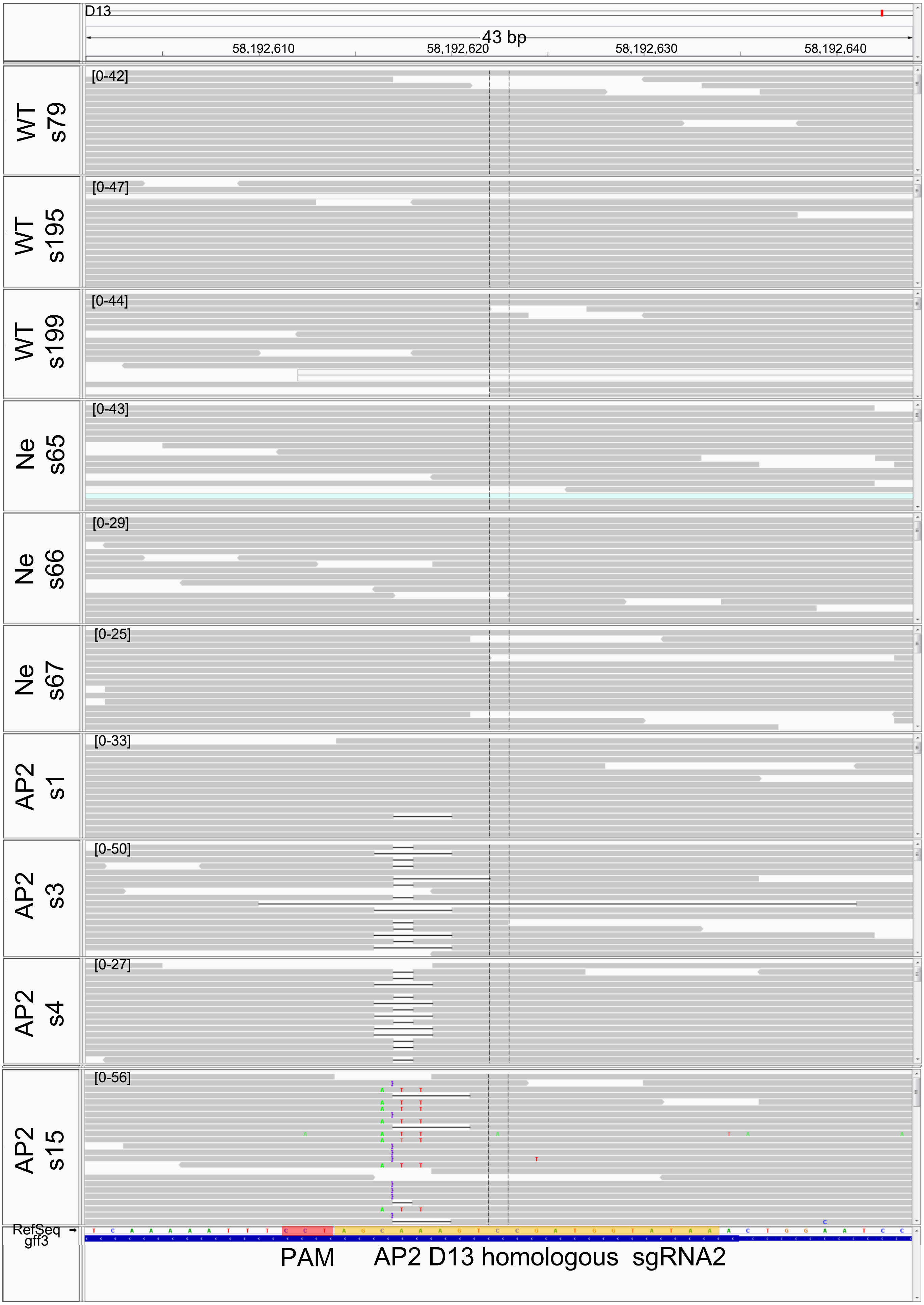

(d) AP2 A12 homologous sgRNA1

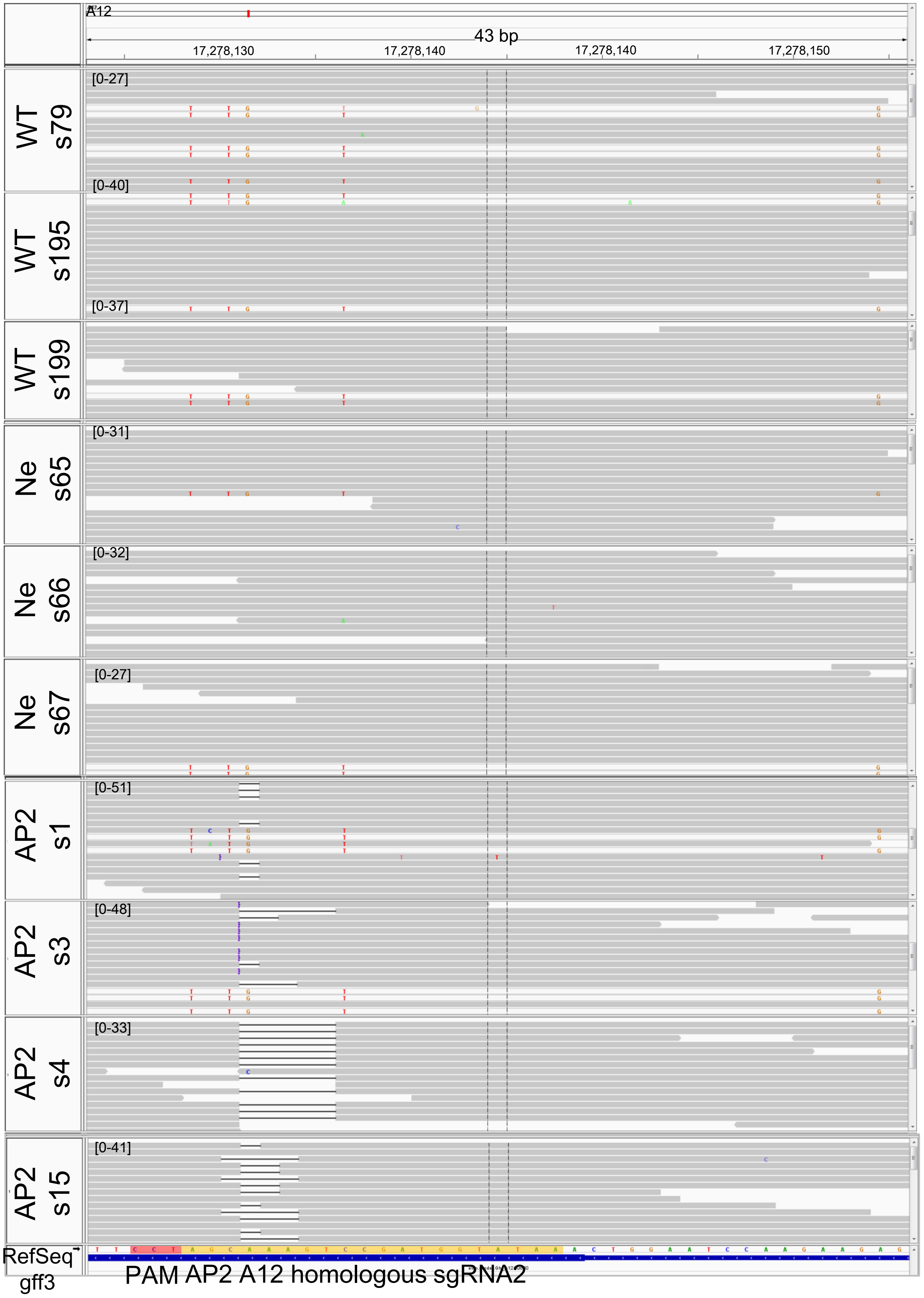

Figure S2 Confirmation of the *AP2* on-target mutation in two sgRNAs from WT, negative and CRISPR/Cas9 plants based on the WGS data. The orange box represents the target sites, and the red box represents the PAM sequence. Whole genome sequencing analysis at sgRNA2 target region of *MYB44* in 3 wild-type, 3 negative plants and 4 CRISPR/Cas9-edited *AP2* plants by Integrative Genomics Viewer (IGV). The number in the square brackets e.g. [0-56] represents the WGS supporting sequence reads with on-target sites and the pileup strip represent Cas9-edited *MYB44* plants a heterozygous deletion of 43-bp of *AP2*. The *AP2* sgRNA1 and sgRNA2 homologous loci reads were showed in (a-b) and (c-d) , respectively.

(a)

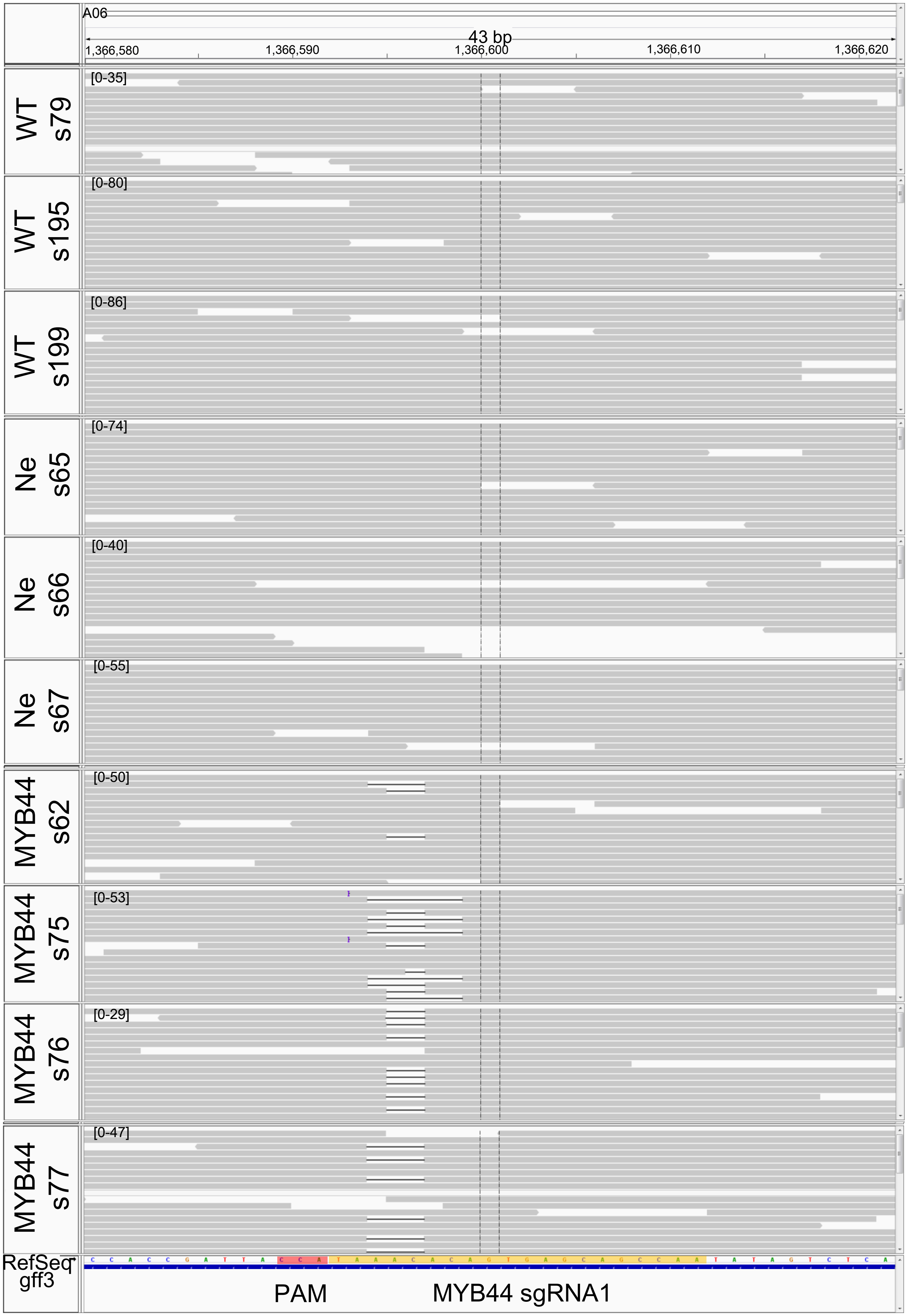
A06
43 bp
1,366,580
1,366,590
1,366,600
1,366,610
1,366,620
WT s79
[0-35]
WT s195
[0-80]
WT s199
[0-86]
Ne s65
[0-74]
Ne s66
[0-40]
Ne s67
[0-55]
MYB44 s62
[0-50]
MYB44 s75
[0-53]
MYB44 s76
[0-29]
MYB44 s77
[0-47]
RefSeq gff3
PAM
MYB44 sgRNA1

(b)

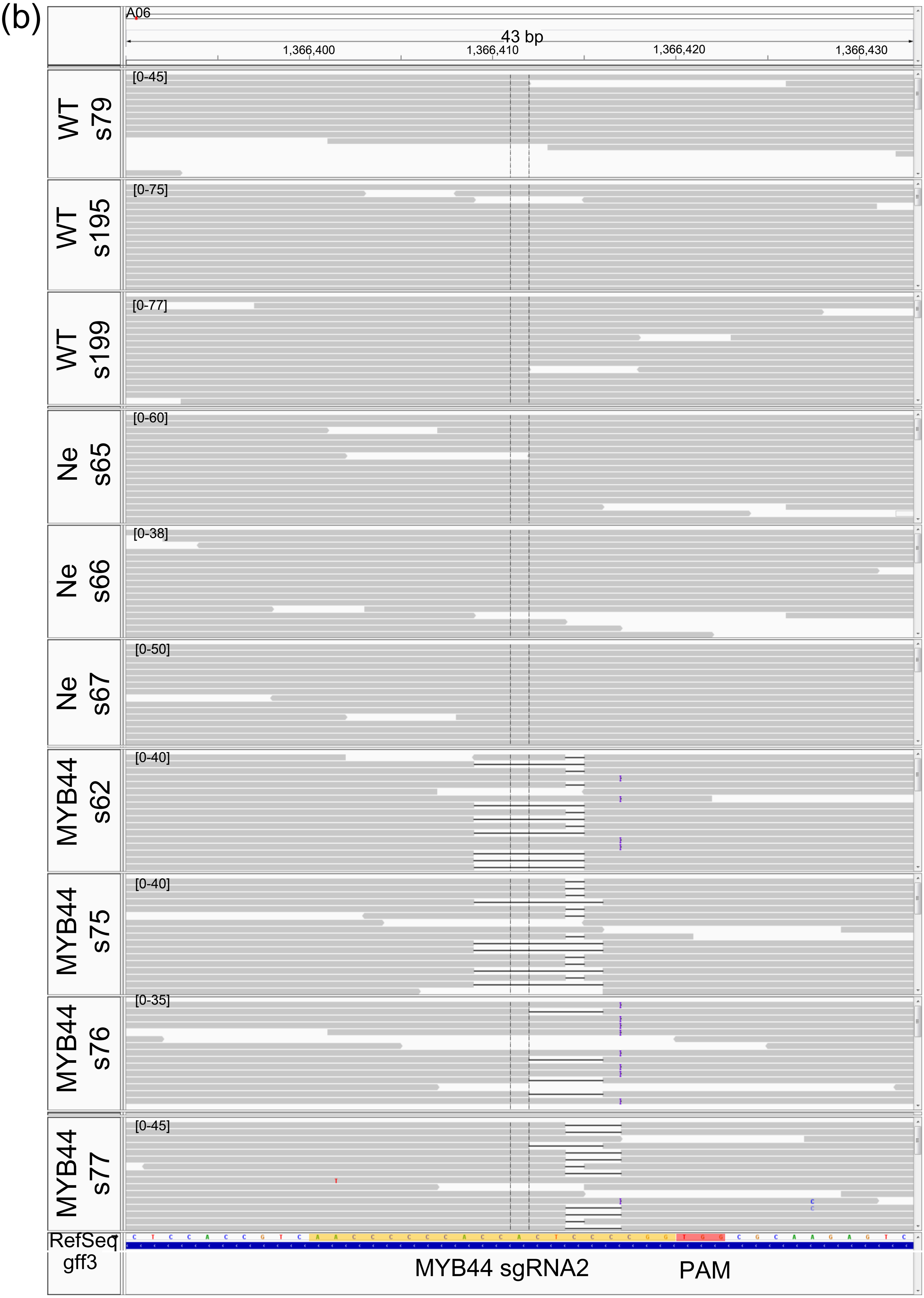

Figure S3 Confirmation of the *MYB44* on-target mutation in two sgRNA loci from WT, negative and CRISPR/Cas9 plants based on the WGS data.

(a) ARC A-genome sgRNA1

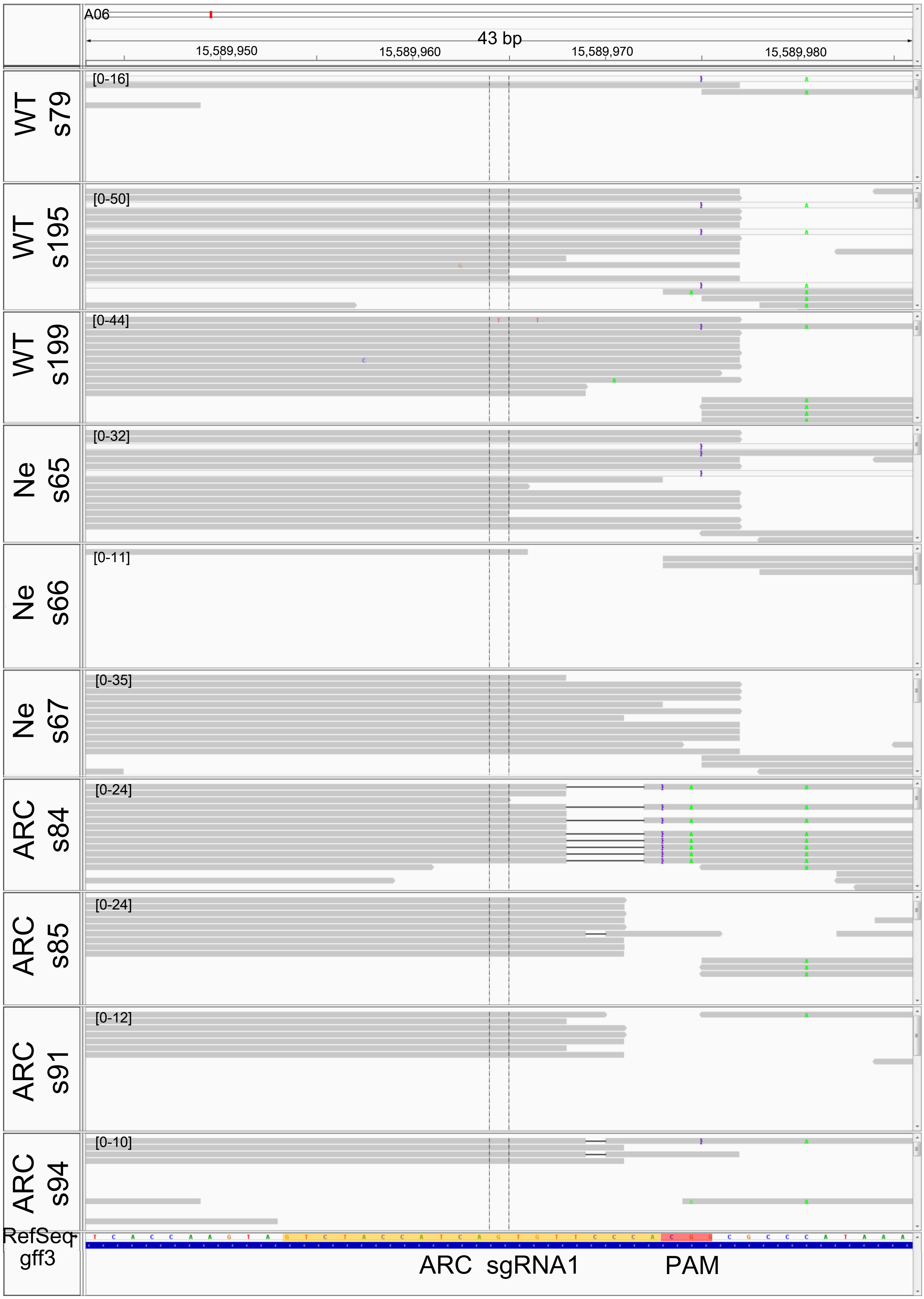

(b) ARC D-genome sgRNA1

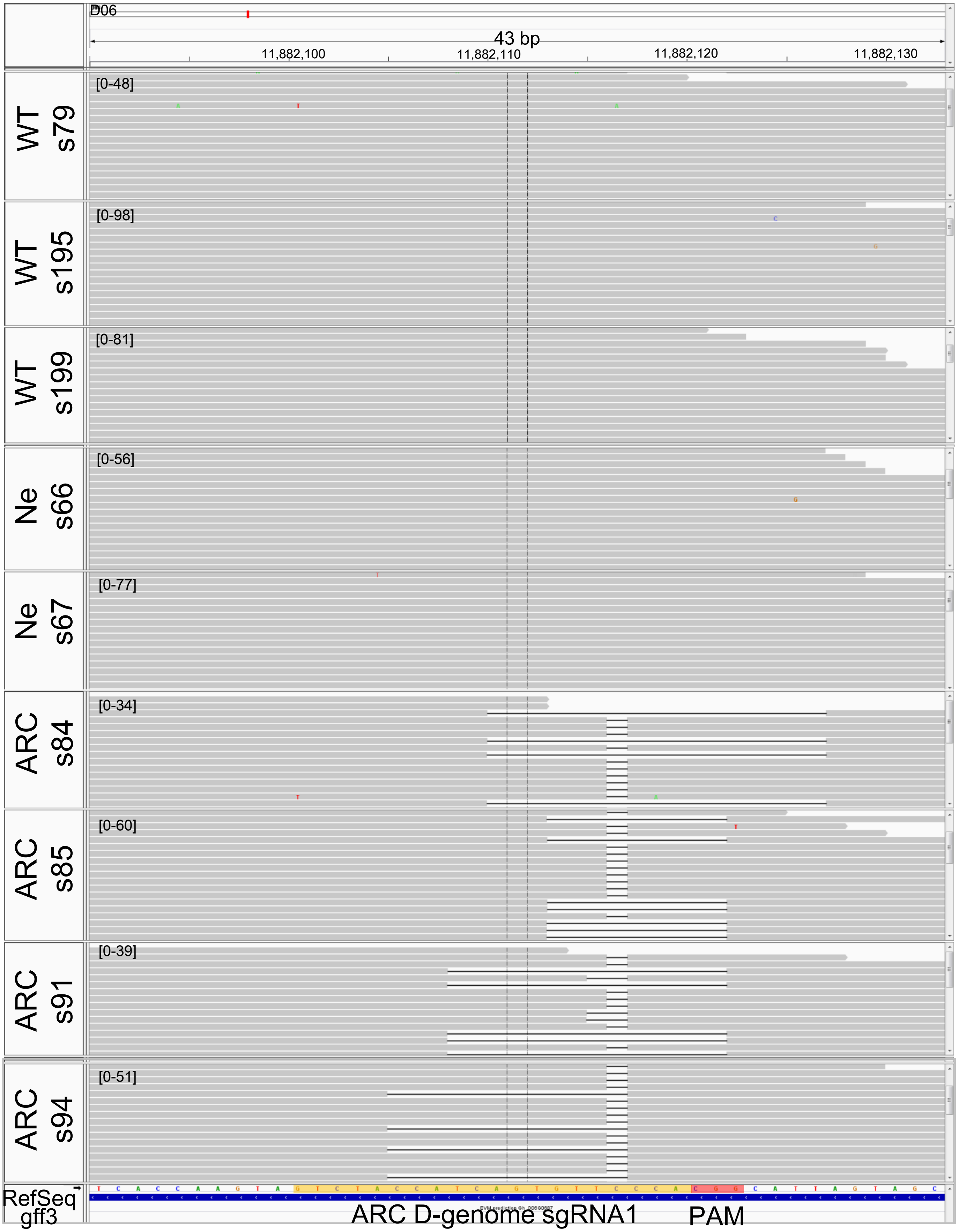

(c) ARC A-genome sgRNA2

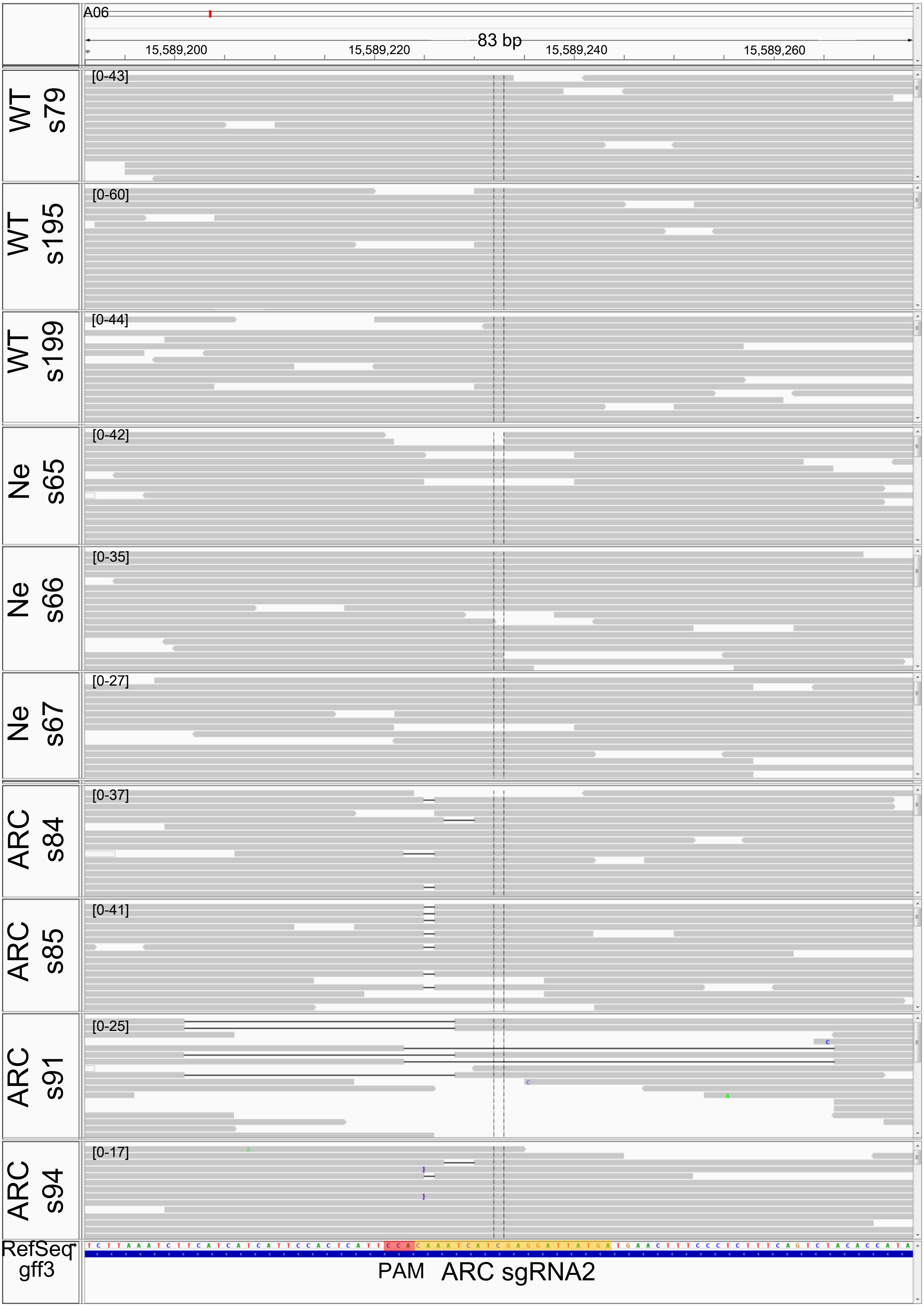

(d) ARC D-genome sgRNA2

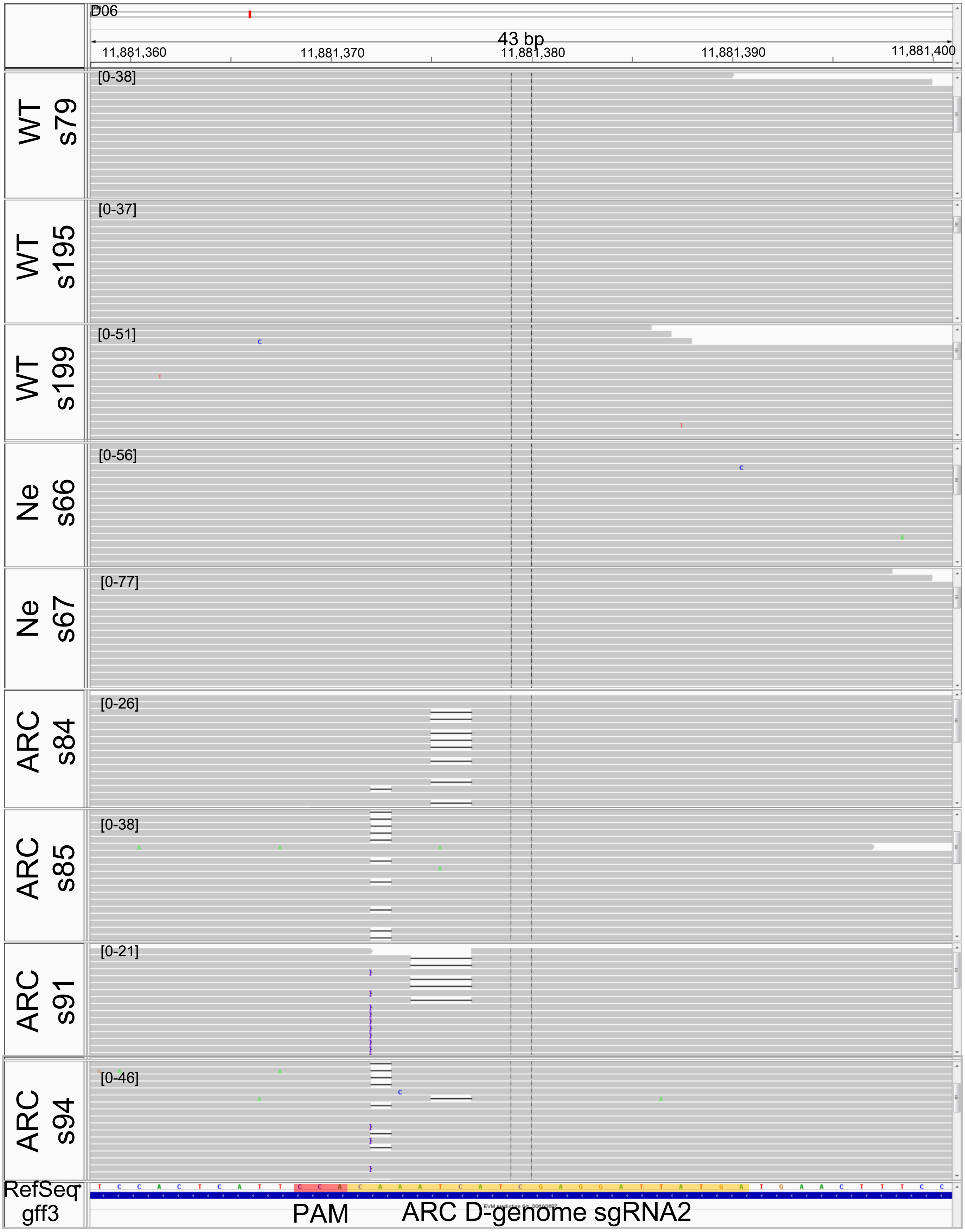

Figure S4 Confirmation of the *ARC* on-target mutation in two sgRNA loci from WT, negative and CRISPR/Cas9 plants based on the WGS data. The *ARC* sgRNA1 and sgRNA2 homologous loci reads were showed in (a-b) and (c-d) , respectively.

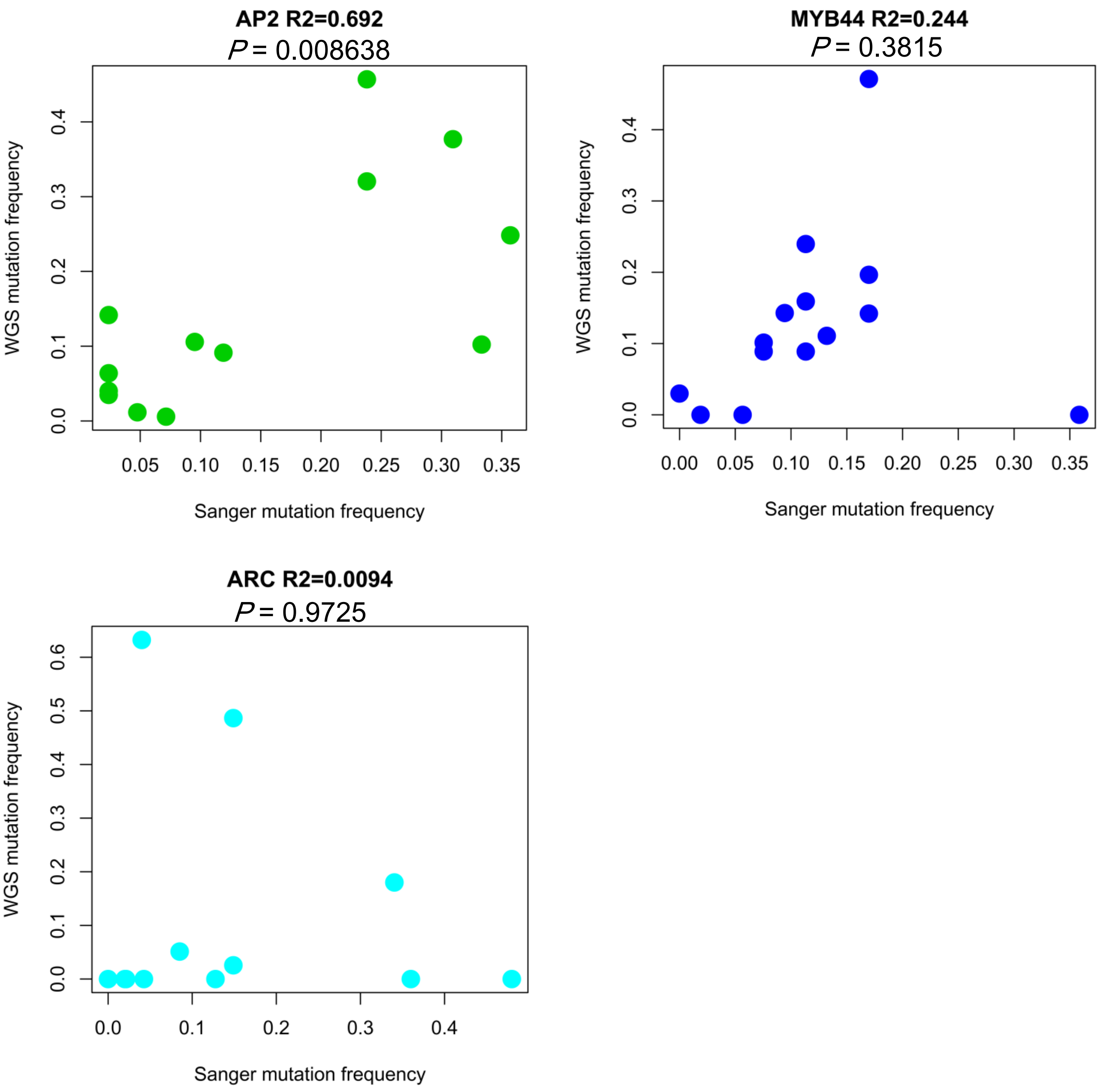

Figure S5 Scatterplot of on-target site correlation of sanger sequencing (x axis) and Whole genome sequencing (y axis). The Correlation Coefficient was calculated based on Pearson’s method (Pearson's correlation coefficient).

Tissue culture process (TSP) mutations in AP2 Cas9-edited plants

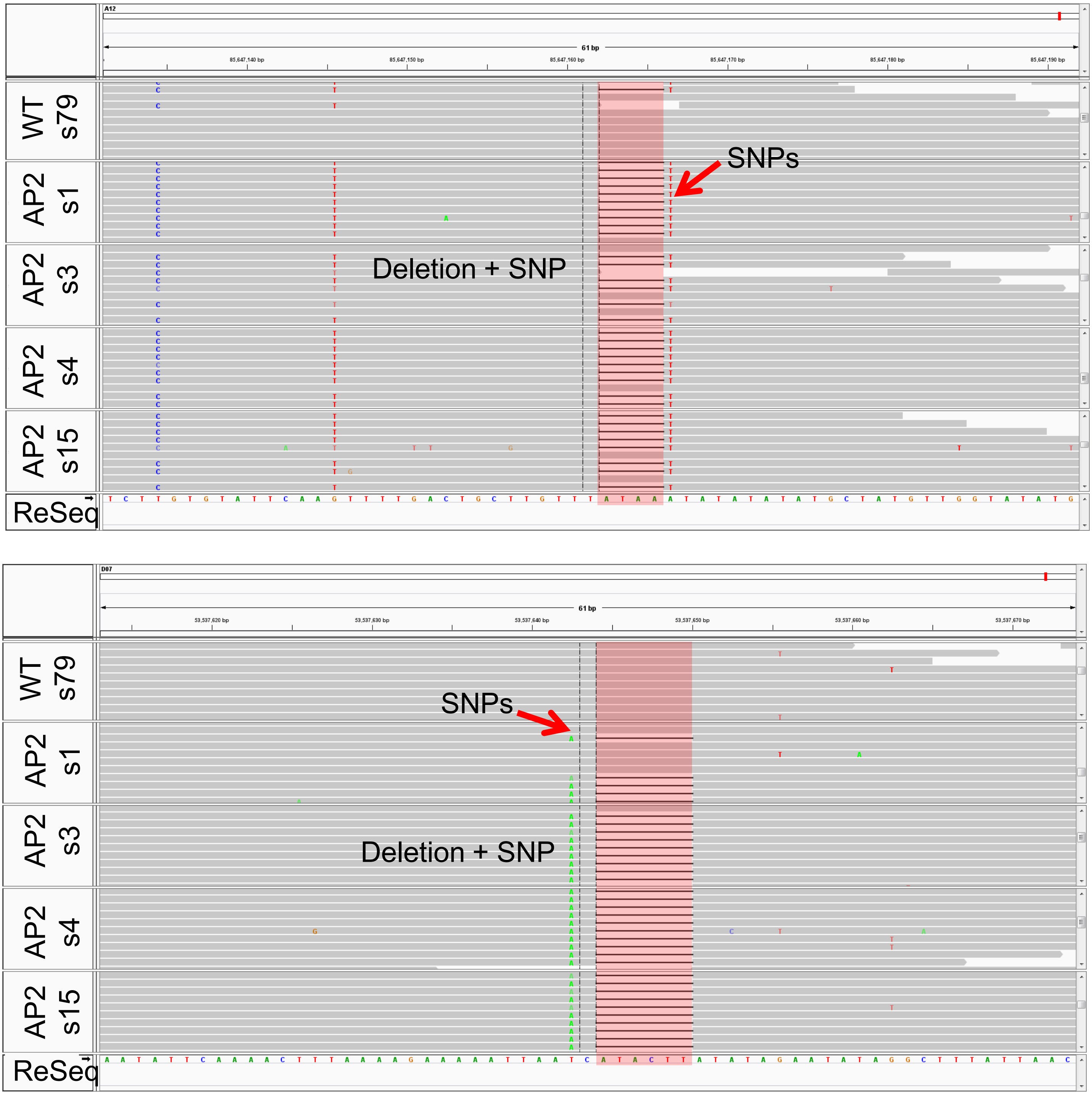

Tissue culture process (TSP) mutations in MYB44 Cas9-edited plants

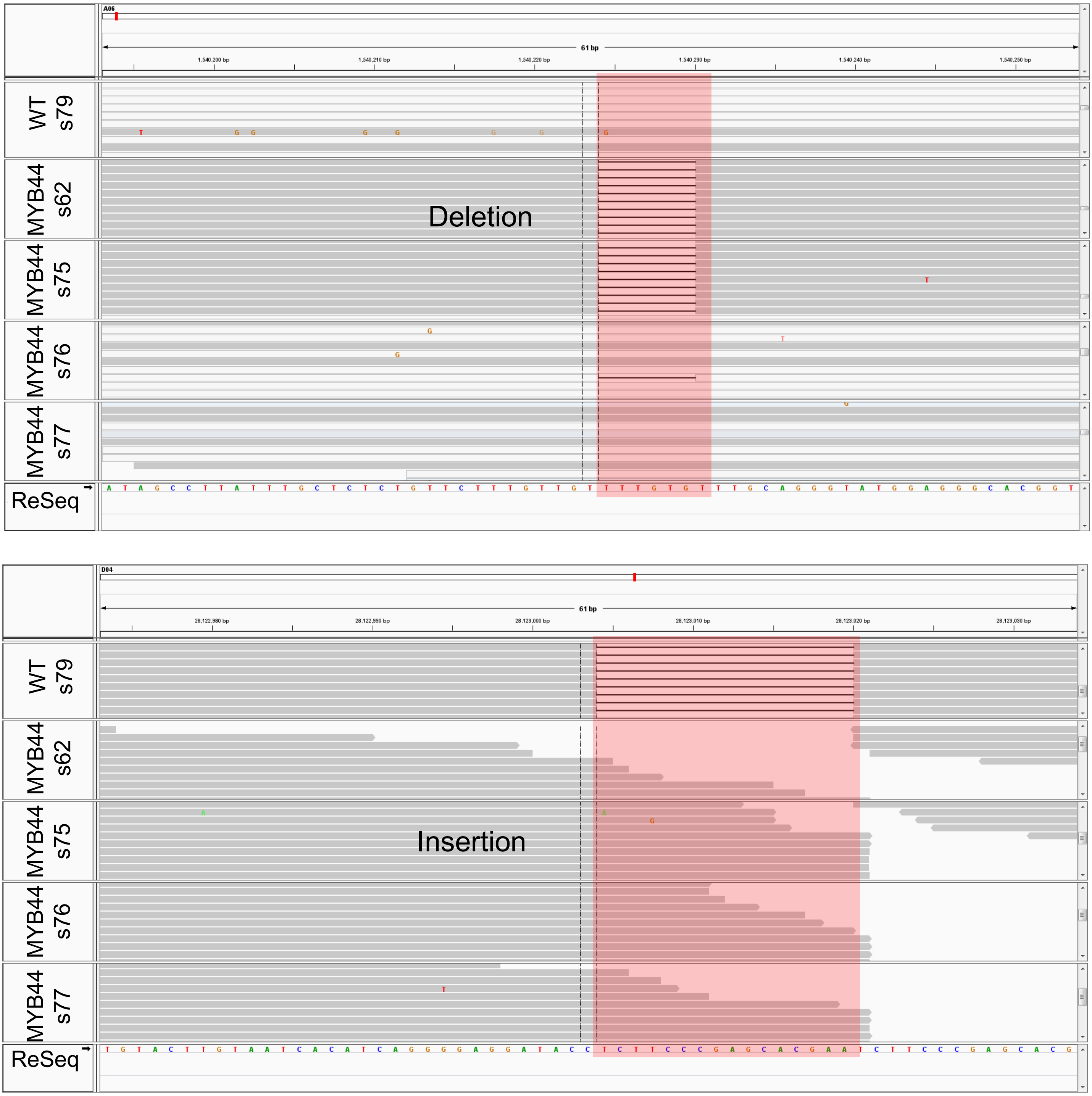

Tissue culture process (TSP) mutations in ARC Cas9-edited plants

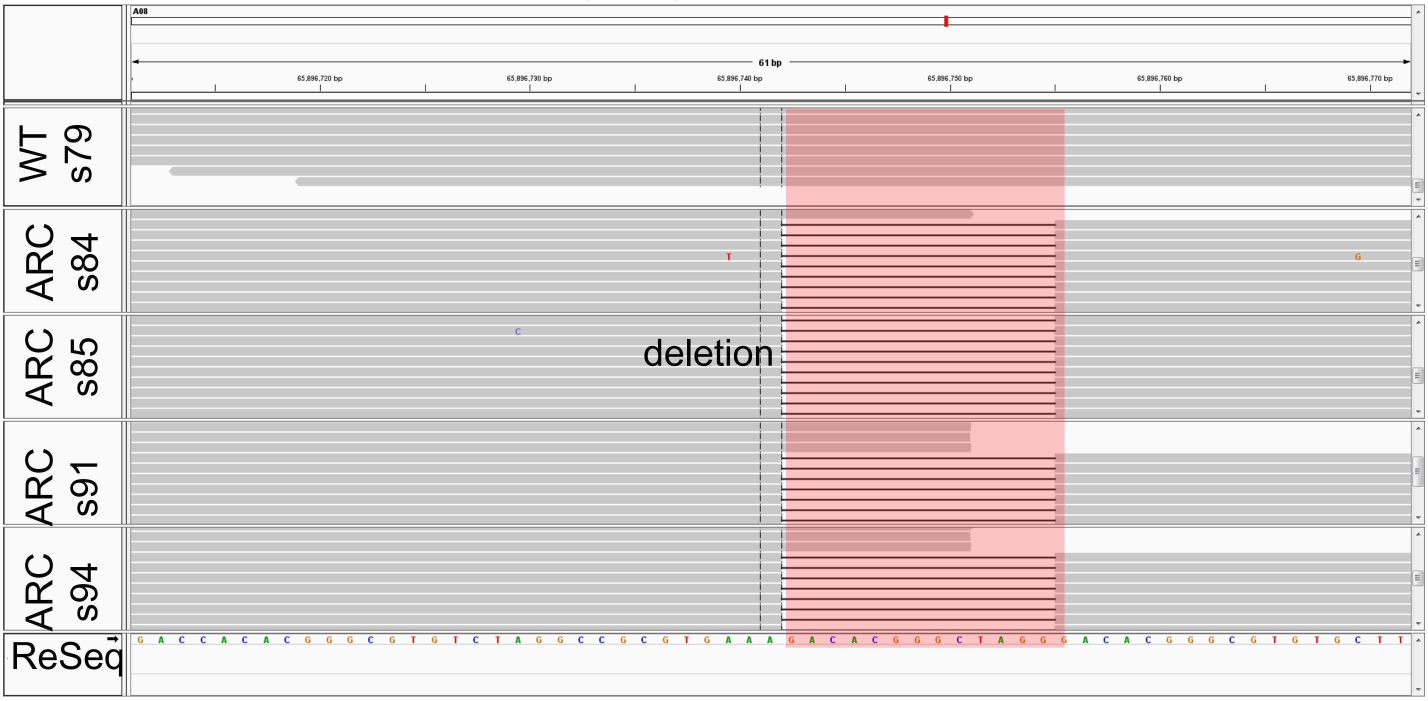

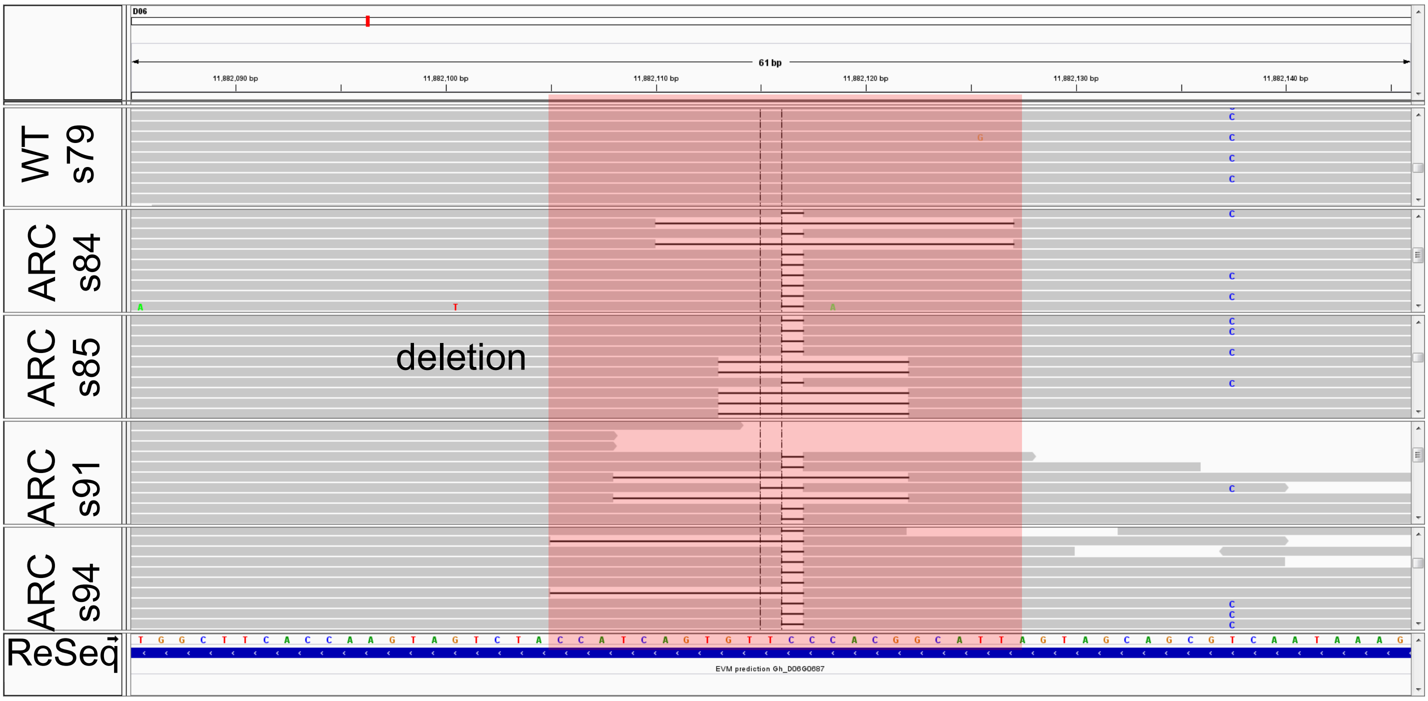

Figure S6 The somaclonal variation or/and inherent genetic variation were detected in *AP*2, *MYB44*, *ARC* Cas9-edited plants and WT by IGV. The red box and arrow represent the SNPs and indels, respectively.

Common and private SNPs in different Cas9-edited lines

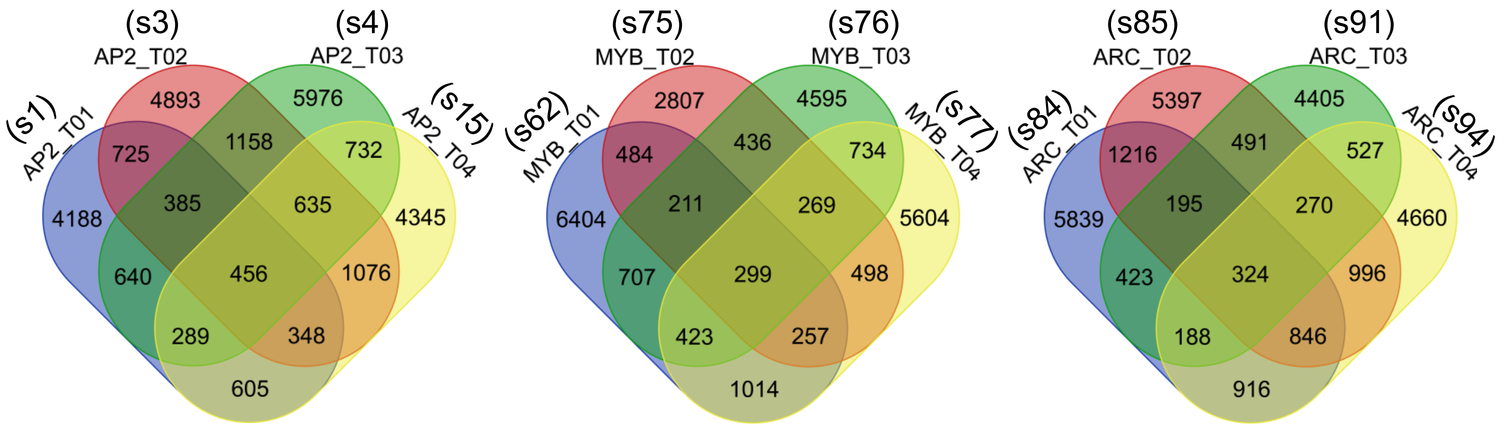

Common and private Indels in different Cas9-edited lines

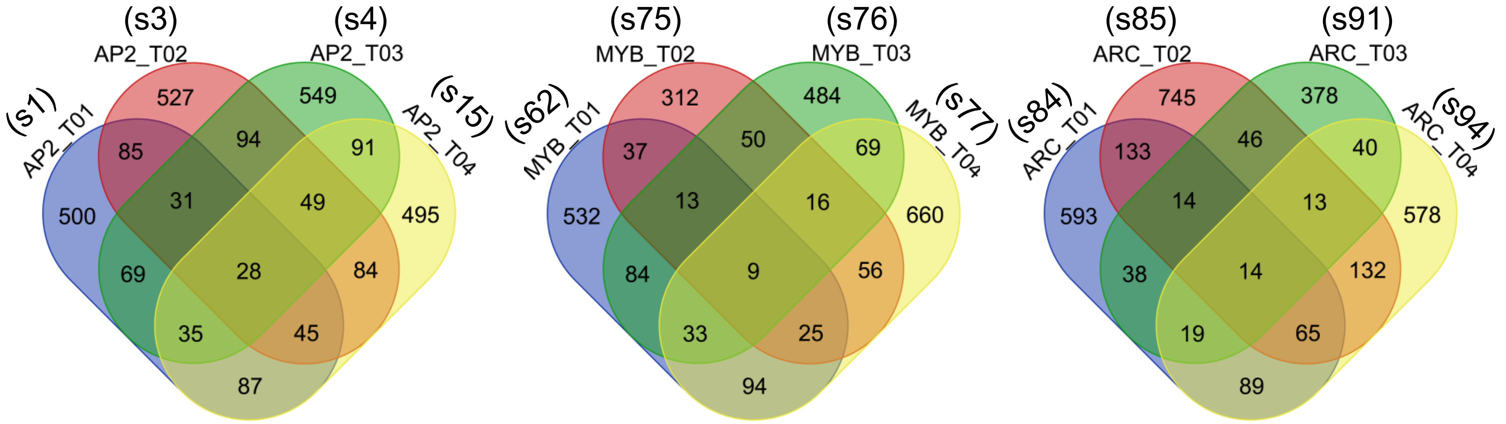

Figure S7 The common and private SNPs/indels in four Cas9-edited lines of *AP2, MYB44, ARC*.

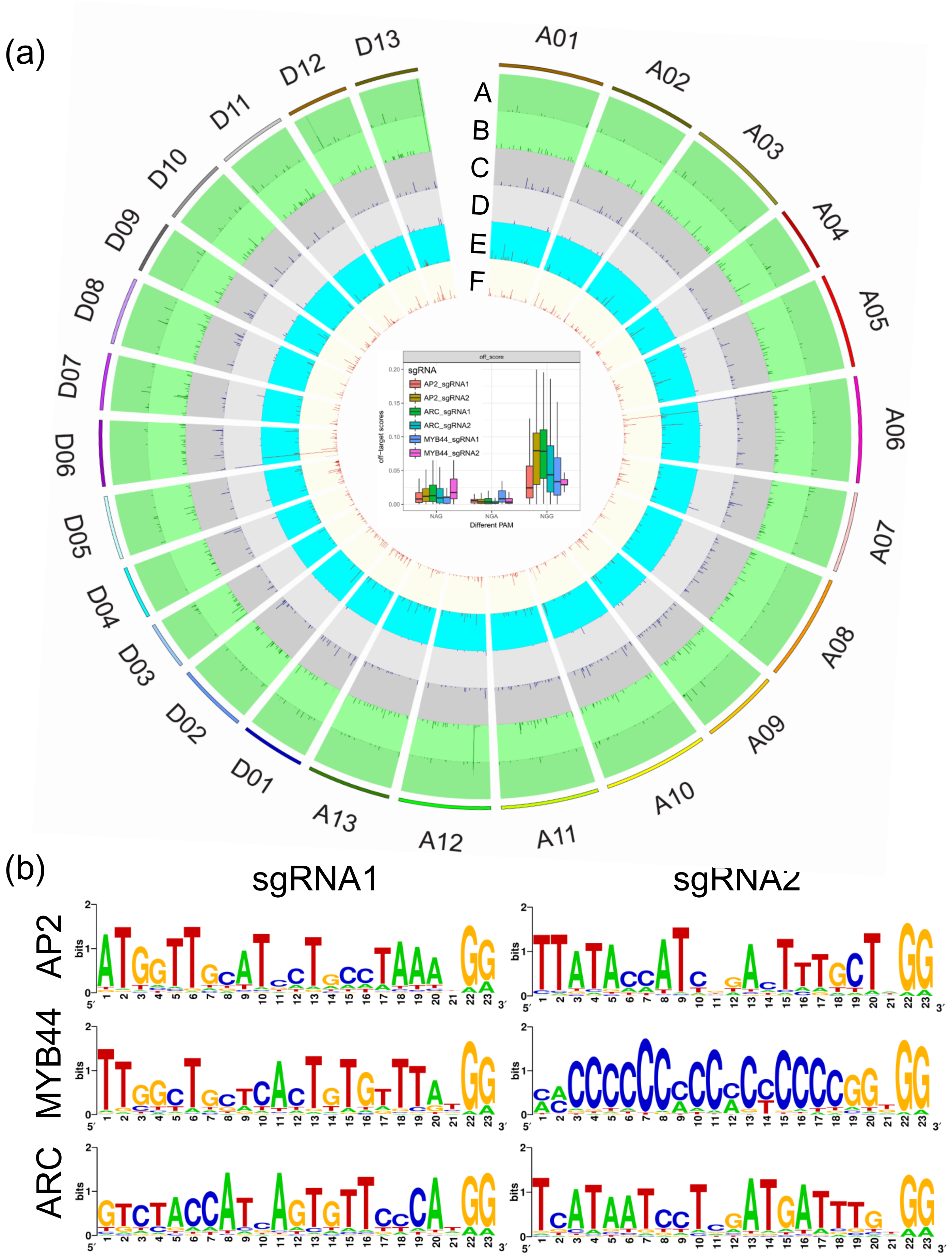

Figure S8 Genome-wide prediction off-target sites. (a) Genome-wide circos plot representing off-target site scores for AP2 sgRNA1 (A) and sgRNA2 (B), MYB44 sgRNA1 (C), and sgRNA2 (D), ARC sgRNA1 (E), and sgRNA2 (F). The different off-target PAM scores were highlighted circos center. (b) The off-target sequences logos were showed.

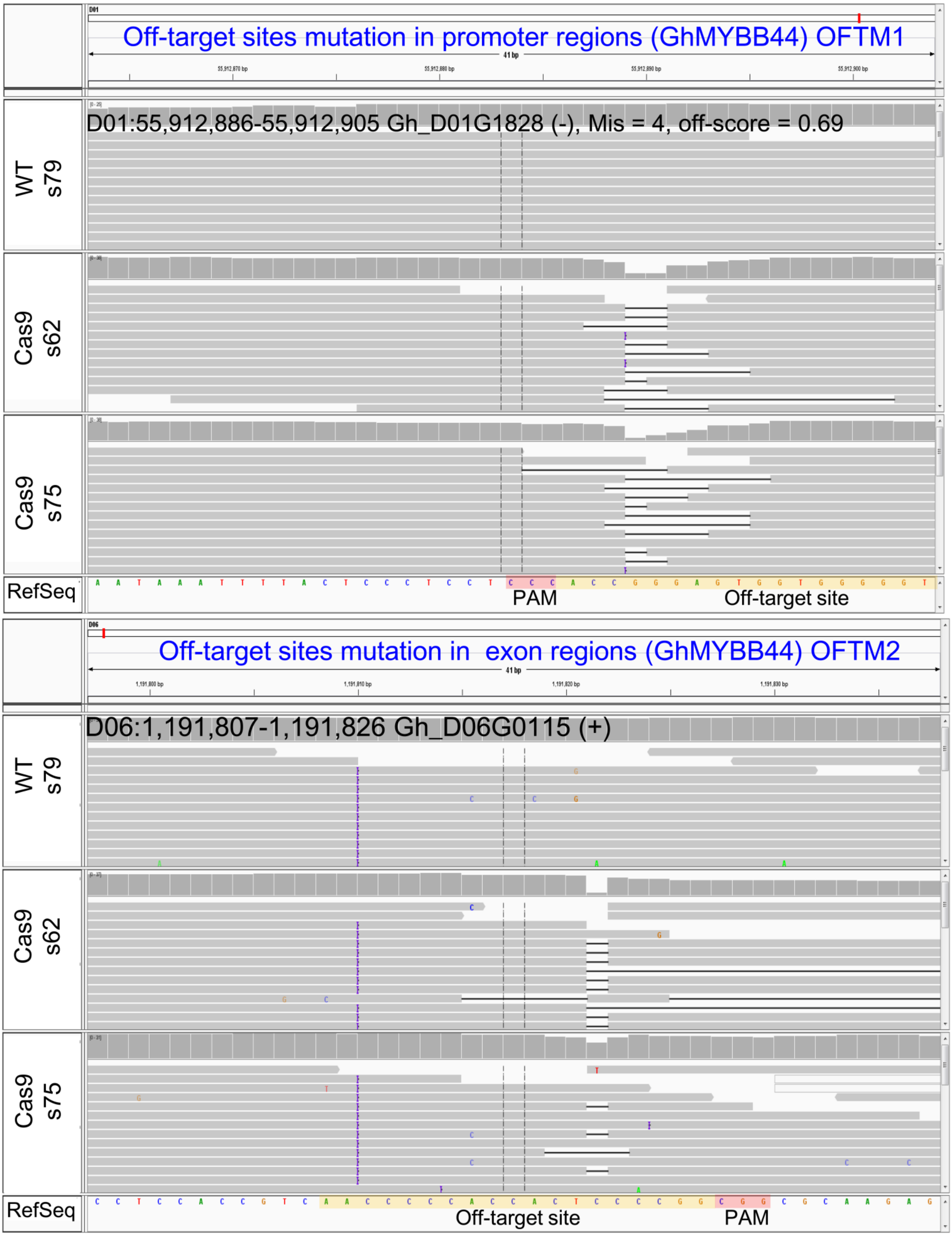

Figure S9 The potential off-target mutations in *MYB44* Cas9-edited cotton plants. The data show the variations in Cas9-s62, Cas9-s75 and WT plants.

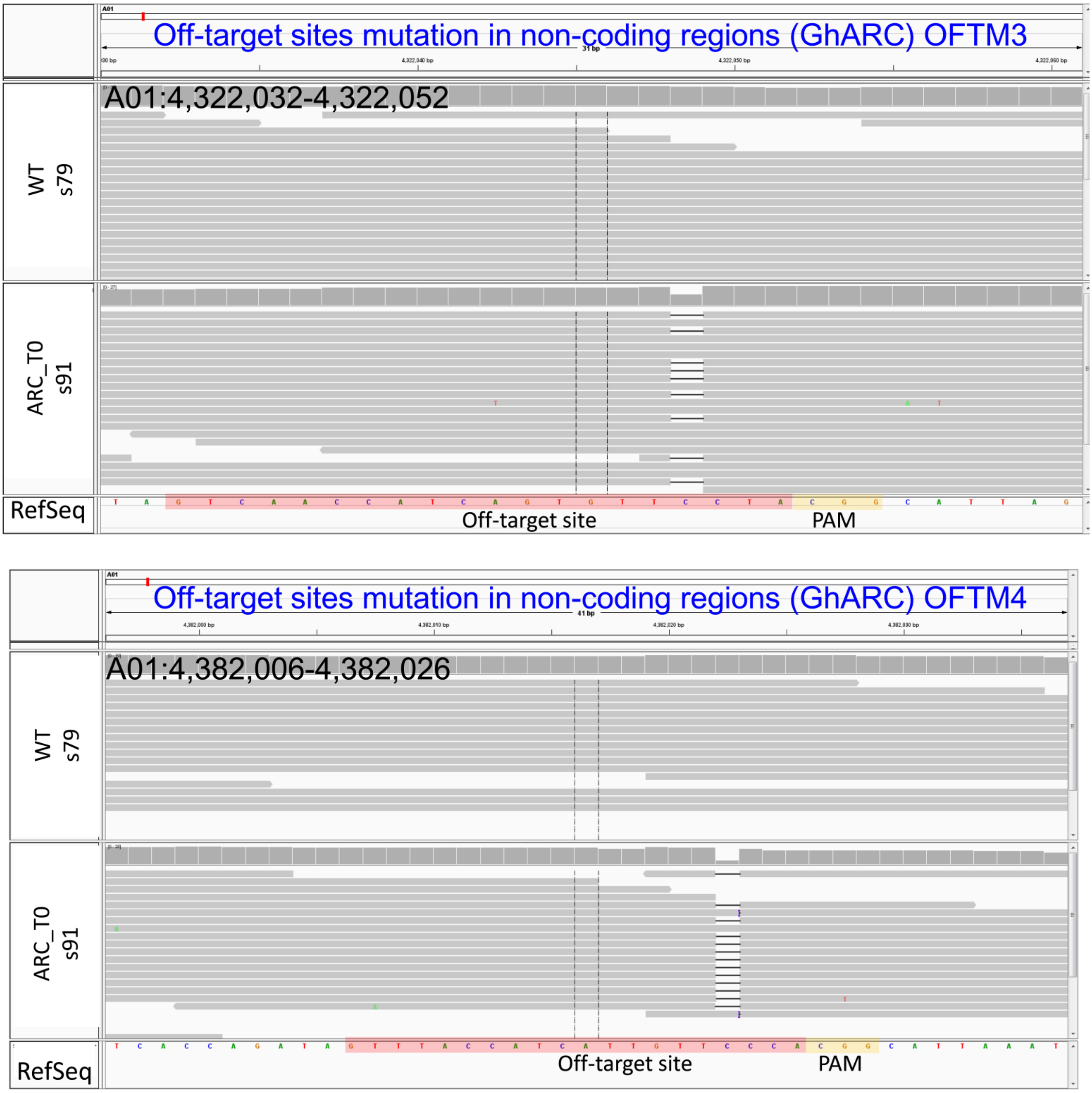

Figure S10 The potential off-target mutations in ARC Cas9-edited cotton plants. The data show the variations in Cas9-s91 and WT Plants.

AP2 sgRNA1 site1 D13: 58,193,326-58,193,348 (Gh_A12G0630)

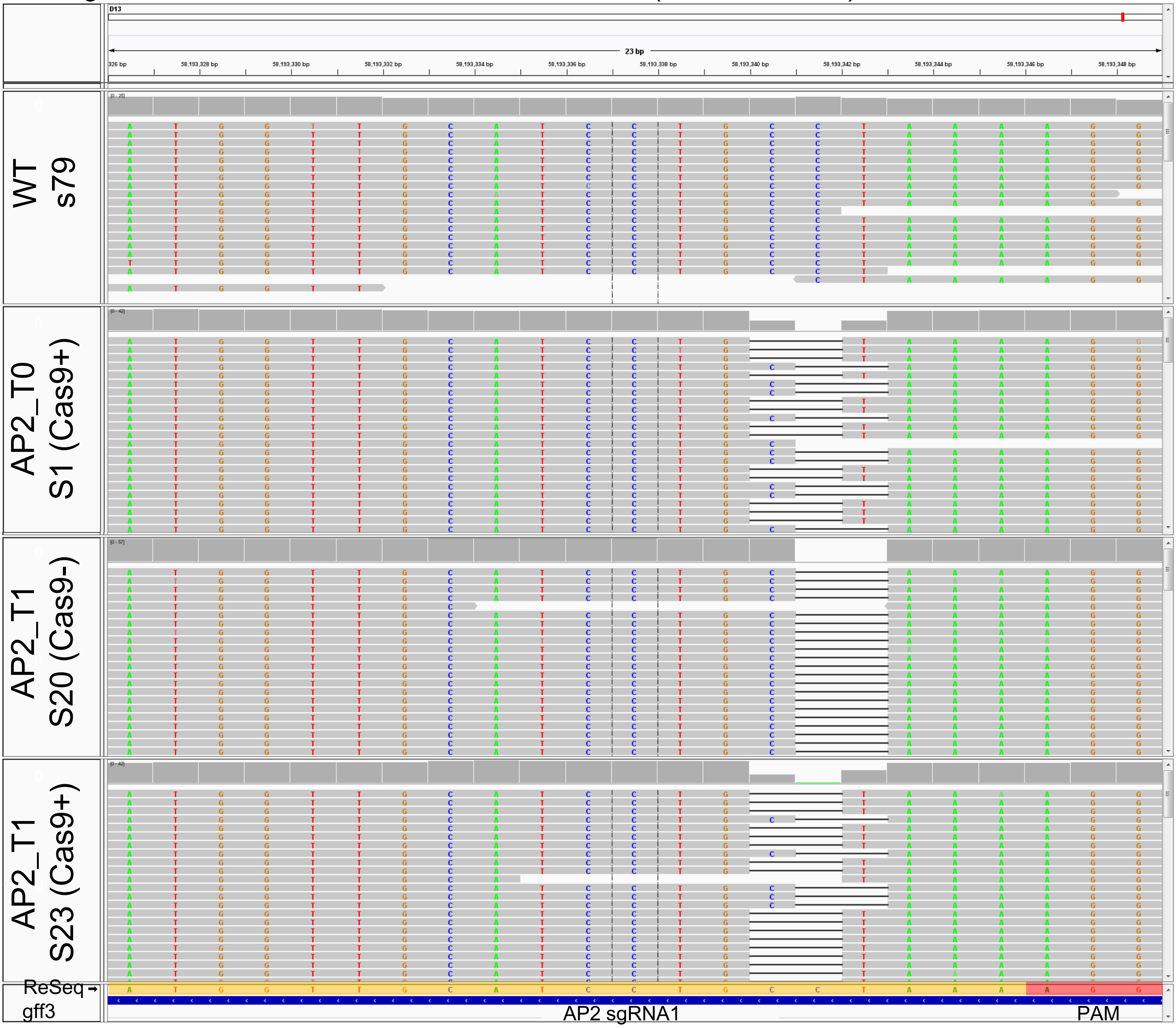

AP2 sgRNA1 site2 D12: 12,897,468-12,897,490 (Gh_D12G0647)

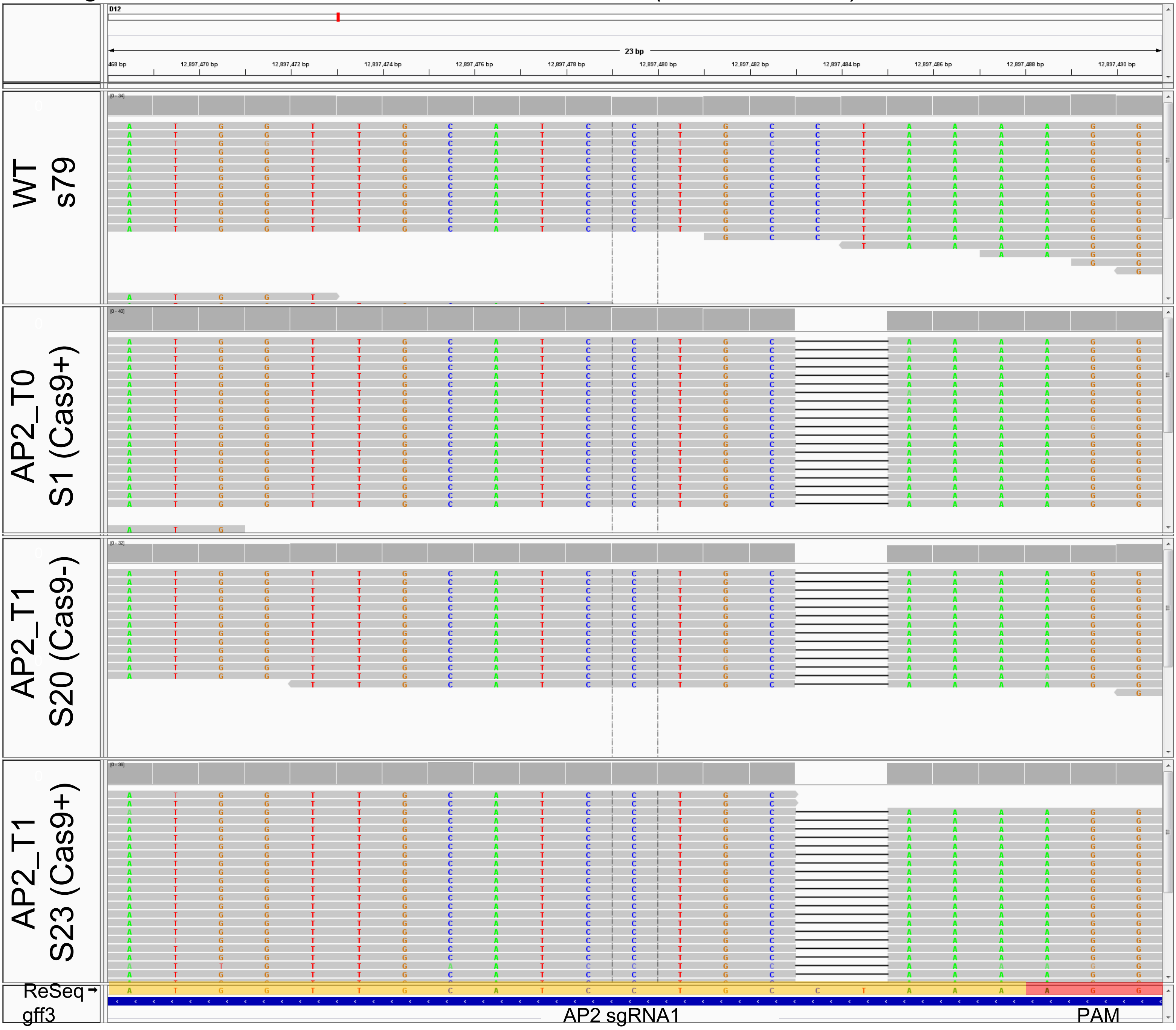

AP2 sgRNA2 site1   D13: 58,192,611-58,192,633 (Gh_D13G2207)

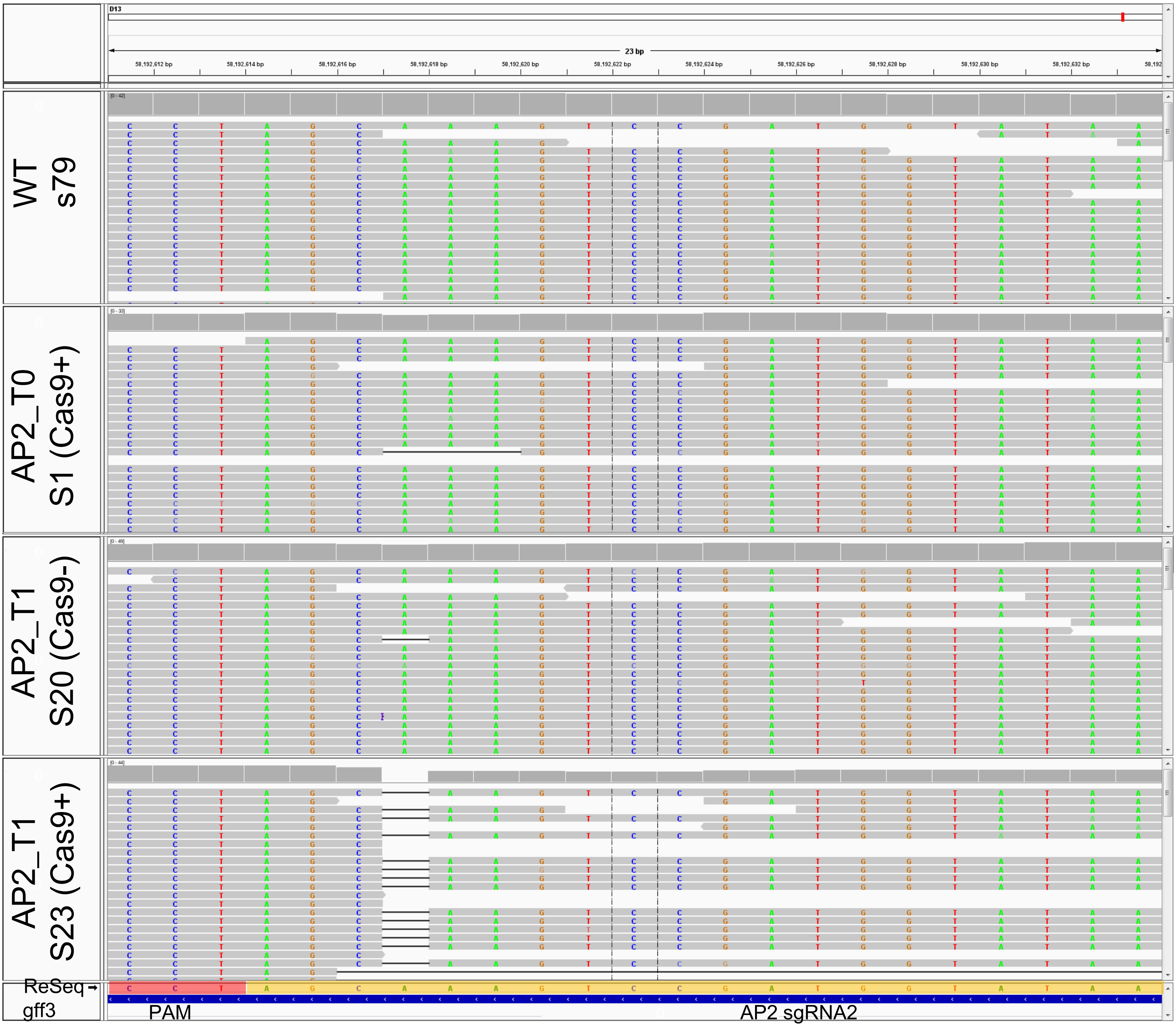

AP2 sgRNA2 site2 A12: 17,278,125-17,278,147 (Gh_A12G0630)

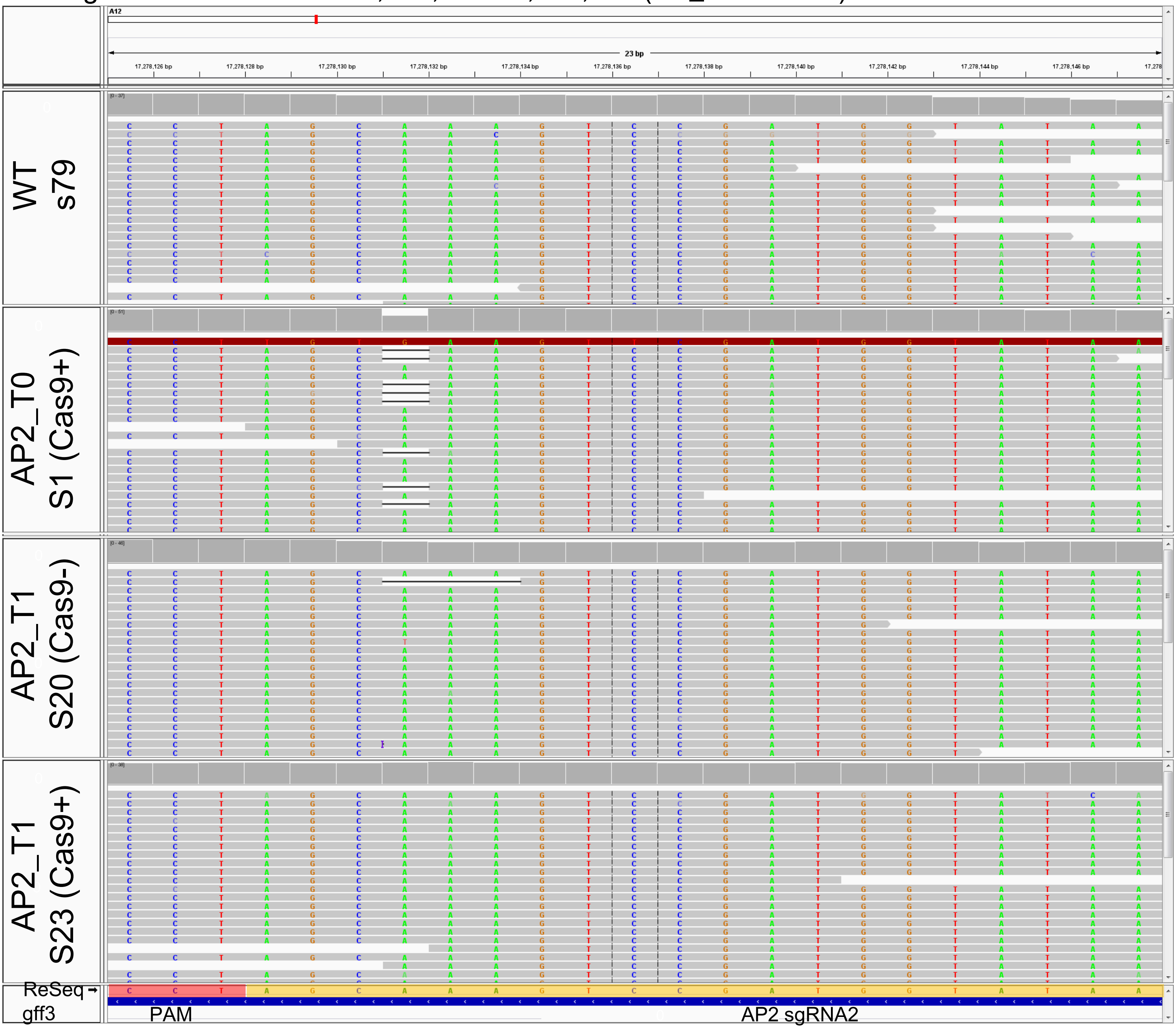

Figure S11 Confirmation of the *AP2* mutations of inheritance from T0 to T1 plants in two sgRNA loci by WGS data. The orange box represents the sgRNA target sites, and the red box represents the PAM sequence.
