# Supplementary material for: Whole genome sequencing reveals rare off‐target mutations and considerable inherent genetic or/and somaclonal variations in CRISPR/Cas9‐edited cotton plants
Source: Plant Biotechnol J. 2018 Oct 30;17(5):858–68. doi: 10.1111/pbi.13020 (PMC6587709; doi:10.1111/pbi.13020)
Supplement: Supplementary file 2 — Table S1 The depth of whole genome sequencing data. Table S2 Summary of target genes in CRISPR/Cas9 editing. Table S3 The different on‐target site mutation frequency of AP2, MYB44, ARC in each target site by Sanger sequencing. Table S4 The summary of variants calling by the SAMtools and GATK. Table S5 Genomic distribution of private variations in Cas9‐edited plants. Table S6 Summary of the off‐target sites across six sgRNA of three target genes. Table S7 Identification of most off‐target site mutations in CRISPR/Cas9 edited plants. Table S8 Summary of new off‐targets and PAMs in WT plants. Table S9 Primers used for on‐targets and off‐targets. [file PBI-17-858-s002.pdf]

Table S1 The depth of whole genome sequencing data.

Lines	Clean reads	Depth
AP2 (s1)	276,317,592	33.16
AP2 (s3)	349,228,878	41.91
AP2 (s4)	241,999,512	29.04
AP2 (s15)	325,942,108	39.11
MYB44 (s62)	270,028,347	32.4
MYB44 (s75)	332,968,819	39.96
MYB44 (s76)	236,182,978	28.34
MYB44 (s77)	299,672,131	35.96
ARC (s84)	307,048,805	36.85
ARC (s85)	343,856,655	41.26
ARC (s91)	323,548,452	38.83
ARC (s94)	214,255,989	25.71
WT (s79)	266,298,091	31.96
WT (s195)	349,729,772	41.97
WT (s199)	305,949,666	36.71
Negative (s65)	267,054,241	32.05
Negative (s66)	265,930,235	31.91
Negative (s67)	256,955,621	30.83
AP2 (s20)	292,204,731	35.06
AP2 (s23)	297,618,345	35.71

Table S2 Summary of target genes in CRISPR/Cas9 editing.

Targets	sgRNA	Chr	start	end	Chr	start	end
AP2	sgRNA1	D13	58193326	58193348	D12	12897468	12897490
AP2	sgRNA2	D13	58192611	58192633	A12	17278125	17278147
MYB44	sgRNA1	A06	1366589	1366611	-	-	-
MYB44	sgRNA2	A06	1366400	1366422	-	-	-
ARC	sgRNA1	A06	15589953	15589975	D06	11882100	11882122
ARC	sgRNA2	A06	15589221	15589243	D06	11881368	11881390

The AP2 sgRNA1 and sgRNA2 have two homologous target loci (At- and Dt-subgenome loci), respectively. ARC sgRNA1 and sgRNA2 have two homologous target loci (At- and Dt-subgenome loci), MYB44 sgRNA1 and sgRNA2 have single loci (At loci).

Table S3 The different on-target site mutation frequency of AP2, *MYB44*, *ARC* in each target site by Sanger sequencing.

AP2 (42)	types	Sanger clone
AP2 sgRNA1 (WT Sequence)		
**CCT**TTTAGGCAGGATGCAACCAT		
Cas9 mutants (s1, s3, s4, s15)		
**CCT**TTTAGGCAGGATGCAACCAT	no	9
**CCT**TTT**A**AGGCAGGATGCAACCAT	i1	10
**CCT**TTTA--CAGGATGCAACCAT	d2	2
**CCT**TTT--GCAGGATGCAACCAT	d2	10
**CCT**TTT----AGGATGCAACCAT	d4	1
**CCT**TTTC---AGGATGCAACCAT	i1+d3	2
**CCT**TTT----AGGACGCAACCAT	s1+d5	1
**CCT**TTTAGGCAGGATGCAACCAT	other	1
**CCT**TCTATAGTGTCACCTAAATAGCT	other	2
TT-------------------GG	d18	4
AP2 sgRNA2 (WT Sequence)		
TTATACCATCGGACTTTGCT**AGG**		
Cas9 mutants (s1, s3, s4,s15)		
TTATACCATCGGACTTTGCT**AGG**	no	7
TTATACCATCGGACTTTGCT**AGA**	s1	2
TTATACCATCGGACT**AAT**CT**AGG**	s3	5
TTATACCATCGGACTTTT**T**GCT**AGG**	i1	5
TTATACCATCGGACT-TGCT**AGG**	d1	11
TTATACCATCGGAC--TGCT**AGG**	d2	3
TTATACCATCGGAC---GCT**AGG**	d3	1
TTATACCATCGGACTTTGCT---	d3	1
TTATACCATCGGAC----CT**AGG**	d4	1
TTATACCATCGG-----GCT**AGG**	d5	2
CTTT-------------GCT**AGG**	d13	1
CTTTCTCATAGCTCACGCTGT**AGG**	other	2
CTTTGCT**AGG**	other	1
TTATACCATCGGACACT**AGG**	other	1
TTATACCATCGGACCT**AGG**	other	1
TTATACCATCGGACGCT**AGG**	other	1
TTATACCATCGGGCT**AGG**	other	2
MYB44 (53)		
MYB44 sgRNA1 (WT Sequence)		
TTGGCTGCTCACTGTGTTTA**TGG**		

TTGGCTGCTCACTG**T-**TTTA**TGG**		
TTGGCTGCTCACTGTGTTTA**TGG**	no	9
TT**A**GCTGCTCACTGTGTTTA**TGG**	s1	1
TTGGC**C**GCTCACGGTGTTTA**TGG**	s1	1
TTGGCTGCTCAC**G**GTGCTTA**TGG**	s1	14
TTGGCTGCTCAC**C**GTGTTTA**TGG**	s1	1
TTGG**C**TGCTCACTGTGCTTA**TGG**	s1	1
TTGGCTGCTCAC**G**GTG**C**TTA**TGG**	s2	1
TTGGCTGCTCACTGT-TTTA**TGG**	d1	1
TTGGCTGCTCACTGTG-TTA**TGG**	d1	1
TTGGCTGCTCACTGT--TTA**TGG**	d2	9
TTGGCTGCTCACTGT---TA**TGG**	d3	7
TTGGCTGCTCACT-----TA**TGG**	d5	4
TTGGCTGCTCCGGTGTTTA**TGG**	other	1
TTGGCTGCTCCTGTTTTA**TGG**	other	1
TTGGTTGCTCATTGTCTTA**TGG**	other	1
MYB44 sgRNA2 (WT Sequence)		
**CCA**CCGGGGAGTGGTGGGGGGTT		
**CCA**CCGGGGAGTGGTGGGGGGTT	no	0
**CCG**CCGGGGAGTGGTGGGGGGTT	s1	3
**CCA**CCG**A**GGGAGTGGTGGGGGGTT	i1	9
**CCA**CCGGGA--GTGGTGGGGGGTT	d2	6
**CCA**CCGA----GTGGTGGGGGGTT	d4	6
**CCA**CCGG-----TGGTGGGGGGTT	d5	4
**CCA**CCGGG-------TGGGGGGTT	d7	5
**CCA**CCGG--------TGGGGGGTT	d8	6
**CCG**CC-GGGAGTGGTGGGGGGTT	s1+d1	6
**CCG**CC**G**GGGGAGTGGTGGGGGGTT	s1+i1	3
**CCG**CC--GG-----TGGGGGGTT	s1+d7	1
**CCA**T**T**CCAAGTGGCGGATCGTT	other	1
**CCG**CCCCGAGCGCCGGATCGTT	other	1
GGTGGGGGGTT	other	2
GTGGGGGGTT	other	1
TGGGGGGTT	other	1
ARC (47)		
ARC sgRNA1 (WT Sequence)		
**CCG**TGGGAACACTGATGGTAGAC		
**CCG**TGGGAACACTGATGGTAGAC	no	0
**CCG**T-GGAACACTGATGGTAGAC	d1	2
---TGGGAACACTGATGGTAGAC	d3	1
**CCGC**GGGAA---T--T**C**G-A-TT	s2+d7	18
**CGT**TGGGA**G**C**T**CT**CCCAT**ATGGT**C**GAC	s4+i5	24

**CGT**TGGGA**G**C**T**CTC**CCAT**ATGGT**C**GAC	s5+i4	1
TAGGGCGAATTGGG	other	1
ARC sgRNA2 (WT Sequence)		
TCATAATCCTCGATGATTTG**TGG**		
TCATAATCCTCGATGATTTG**TGG**	no	7
TCATA**G**TCCTCGATGATTTG**TGG**	s1	1
TCATAATCCTCGATGA**T**TTTG**TGG**	i1	4
TCATAATCCTCGATGATT-G**TGG**	d1	16
TCATAATCCTCGATG-TTTG**TGG**	d1	1
TCATAATCCTCGAT--TTTG**TGG**	d2	6
TCATAATCCTCGA---TTTG**TGG**	d3	7
TCA**C**AATCCTCGATGA**T**TTTG**TGG**	s1+i1	1
TCATAATCCTCGA**C**GA**T**TTTG**TGG**	s1+i1	2
TCATAATCCTCGATC--TTG**TGG**	s1+d2	1

Blue and red font represent sgRNA sequences and PAM sequences, respectively. The 'd' represent the deletion genotype. 'i' represent the insertion genotype. 's' represent the substitute genotype. 'other' represent the complex mutation genotype, these mutations may not be caused by Cas9 nucleases. Black fonts with horizontal lines, blue fonts, and red fonts represent the insertion base, mutation base, and PAMs, respectively. The '-' symbol represent the deletion base.

Table S4 The summary of variants calling by the Samtools and Genome Analysis Toolkit (GATK) software.

SNPs

samples	Samtools	GATK	comm	concordance
WT (s79)	3,155,619	3,930,853	3,078,651	1,211,622
WT (s195)	3,120,442	3,858,349	3,018,462	1,214,683
WT (s199	3,102,776	3,773,930	2,983,787	1,210,509
Negative (s65)	3,199,941	3,989,704	3,111,625	1,203,206
Negative (s66)	3,104,199	3,761,514	2,981,547	1,217,124
Negative (s67)	3,084,089	3,730,645	2,953,171	1,209,155
AP2 (s1)	3,088,928	3,850,277	3,028,876	1,266,777
AP2 (s3)	3,076,586	3,866,424	3,025,629	1,284,258
AP2 (s4)	3,054,148	3,759,271	2,987,931	1,270,814
AP2 (s15)	3,083,515	3,854,856	3,024,844	1,260,704
AP2 (s20)	3,078,241	3,841,130	3,018,440	1,262,125
AP2 (s23)	3,069,588	3,818,970	3,007,609	1,258,810
MYB44 (s62)	3,060,180	3,780,173	2,994,620	1,259,373
MYB44 (s75)	3,113,850	3,943,573	3,059,551	1,268,973
MYB44 (s76)	3,082,260	3,834,140	3,020,862	1,261,093
MYB44 (s77)	3,085,656	3,853,163	3,023,995	1,261,356
ARC (s84)	3,129,296	3,953,628	3,068,534	1,269,877
ARC (s85)	3,099,634	3,937,663	3,048,233	1,277,072
ARC (s91)	3,054,742	3,740,965	2,972,339	1,245,699
ARC (s94)	3,112,696	3,930,615	3,055,514	1,264,623

indels

samples	Samtools	GATK	comm	concordance
WT (s79)	294,466	283,343	226,996	149,327
WT (s195)	284840	266119	219544	152,535
WT (s199)	276963	253506	209603	148,567
Negative (s65)	291,586	281,447	224,578	149,636
Negative (s66)	275,889	252,824	208,968	148,842
Negative (s67)	260,513	229,865	189,208	135,845
AP2 (s1)	298,589	285,855	230,681	150,156
AP2 (s3)	303,015	290,344	235,255	150,421
AP2 (s4)	291,579	276,697	223,999	150,405
AP2 (s15)	299,573	286,401	231,462	149,367
AP2 (s20)	299,811	285,347	230,904	149,979
AP2 (s23)	297,557	284,215	229,611	149,834
MYB44 (s62)	290,422	277,896	224,805	149,594
MYB44 (s75)	304,259	291,801	235,130	150,803
MYB44 (s76)	296,587	278,385	225,132	150,637
MYB44 (s77)	299,411	285,137	229,841	150,166
ARC (s84)	299,882	288,462	231,667	151,413
ARC (s85)	303,706	289,113	233,555	149,570
ARC (s91)	290,667	257,004	209,034	145,824
ARC (s94)	304,804	290,772	233,901	149,644

Table S5 Genomic distribution of private variations in Cas9-edited plants.

Cas9-edited plants	#No. total SNPs	#No. total indels	#No.SNPs /indels	#No. intronic	#nonsynonymous SNPs	#No. synonymous SNPs	#No. frameshift indels	#No. nonframe shift indels
AP2 (s1)	4,188	500	240/9	308/51	133	104	4	3
AP2 (s3)	4,893	527	183/8	427/53	103	76	5	3
AP2 (s4)	5,976	549	273/9	546/55	158	112	6	3
AP2 (s15)	4,345	495	128/6	288/50	64	63	3	3
MYB44 (s62)	6,404	532	233/14	456/46	111	103	8	6
MYB44 (s75)	2,807	312	110/5	174/19	63	45	4	1
MYB44 (s76)	4,595	484	201/2	385/66	108	89	1	1
MYB44 (s77)	5,604	660	229/7	361/59	118	98	3	2
ARC (s84)	5,839	593	349/12	520/46	188	150	4	6
ARC (s85)	5,397	745	202/2	392/67	108	83	1	0
ARC (s91)	4,405	378	229/7	431/62	123	105	5	2
ARC (s94)	4,660	578	212/4	322/57	110	85	4	0

All these variations (Total SNPs/indels) were indentified after alignment the genome sequences of CRISPR Cas9-edited plants with WT, negative plants and TM-1 reference genome. These variations in three WT plants has same genotype as three negative plants, but differs from each Cas9-edited plants, which variants called 'total variantion' (depth > 20). Total variations (including tissue culture variations, Cas9-edited variations and pre-existing/inherent variations) were annotated by ANNOVAR software and further analyzed off-target site mutations. Total and private SNPs/indels correspond to **Table S4.**

Table S6 Summary of the off-target sites across 6 sgRNA of three target genes.

Target Gene	sgRNA	No.of mismatch	No.of NGG sites	No.of NAG sites	No.of NGA sites
AP2	sgRNA1	0	2	0	0
		1	0	0	0
		2	2	0	0
		3	6	0	0
		4	33	23	72
		5	398	34	83
	sgRNA2	0	2	0	0
		1	1	0	1
		2	0	0	0
		3	7	0	0
		4	44	24	38
		5	711	31	44
MYB44	sgRNA1	0	1	0	0
		1	1	0	0
		2	0	0	0
		3	1	0	0
		4	39	27	55
		5	641	28	96
	sgRNA2	0	1	0	0
		1	0	0	0
		2	1	0	0
		3	3	0	0
		4	20	6	9
		5	157	2	6
ARC	sgRNA1	0	2	0	0
		1	0	0	0
		2	5	0	0
		3	2	0	0
		4	30	32	26
		5	302	34	28
	sgRNA2	0	2	0	0
		1	0	0	0
		2	7	0	0
		3	14	3	0
		4	131	79	99
		5	730	87	150

The sgRNA sequences alignment were aligned TM-1 reference genome with mismatch (K=0,1,2,3,4,5), the NGG, NAG, NGA were identified from 6 sgRNA sequence.

Table S7 Identification of most off-target site mutations in CRISPR-Cas9 edited plants.

sgRNA	chr	start	end	on-target seq	off-target seq	strand	Mis	off_score	region
AP2 sgRNA1	D12	12822662	12822681	ATGGTTGCATCCTGCCTAAA**AGG**	ATGGTTGCATCTTGCCCAAAAGG	+	2	0.3801	intergenic
AP2 sgRNA1	D11	2751318	2751337	ATGGTTGCATCCTGCCTAAAAGG	CTCATTGCAACCTGCCTAAAGGG	-	4	0.2543	intergenic
AP2 sgRNA1	A12	17258789	17258808	ATGGTTGCATCCTGCCTAAAAGG	GTGGTTGCATCTTGCCCATAAGG	+	4	0.2047	intergenic
AP2 sgRNA1	A09	69549317	69549336	ATGGTTGCATCCTGCCTAAAAGG	ATGGTTACATCCTTCATAAATGG	+	3	0.2667	intergenic
AP2 sgRNA1	D09	44586497	44586516	ATGGTTGCATCCTGCCTAAAAGG	ATGGTTACATCCTTCACAAATGG	+	4	0.1882	intergenic
AP2 sgRNA1	A12	17278848	17278867	ATGGTTGCATCCTGCCTAAAAGG	ATGGTTGCGTCCTGCCTATAAGG	+	2	0.359	Gh_A12G0630
AP2 sgRNA1	A03	98326096	98326115	ATGGTTGCATCCTGCCTAAAAGG	ATGCTTACTTACTGCATAAATGG	+	5	0.2382	Gh_A03G1709
AP2 sgRNA1	A09	61347280	61347299	ATGGTTGCATCCTGCCTAAAAGG	ATGGGTGAATCCTACACAAATGG	+	5	0.2202	Gh_A09G1072
AP2 sgRNA1	D02	33199969	33199988	ATGGTTGCATCCTGCCTAAAAGG	TCAGATGCATCTTGCCTAAAAGG	-	5	0.1731	Gh_D02G1133
AP2 sgRNA1	D01	23350527	23350546	ATGGTTGCATCCTGCCTAAAAGG	ATGCTTGTATCTAGCATAAATGG	+	5	0.1727	Gh_D01G1114
AP2 sgRNA2	A04	43872229	43872248	TTATACCATCGGACTTTGCTAGG	TTATCCCAATAGACTTTGCTGGG	+	4	0.437	Intergenic
AP2 sgRNA2	D03	12150785	12150804	TTATACCATCGGACTTTGCTAGG	TCATACCATCAGGCATTGCTTGG	-	4	0.3236	Intergenic
AP2 sgRNA2	A13	79889925	79889944	TTATACCATCGGACTTTGCTAGG	CTATACCCTAAGACTTTGCTTGG	+	4	0.3184	Intergenic
AP2 sgRNA2	D07	23406425	23406444	TTATACCATCGGACTTTGCTAGG	TTAGAAAATCGGACTTTTCTGGG	-	4	0.2902	Intergenic
AP2 sgRNA2	D08	48109735	48109754	TTATACCATCGGACTTTGCTAGG	TTGTACTATCAGACTTAGCTCGG	+	4	0.2648	Intergenic
AP2 sgRNA2	D07	22204606	22204625	TTATACCATCGGACTTTGCTAGG	CTACATCATCAGATTTTGCTTGG	-	5	0.3777	Gh_D07G1374
AP2 sgRNA2	A11	65031770	65031789	TTATACCATCGGACTTTGCTAGG	TCTTACCACCAGACTTTGCATGG	+	5	0.3142	Gh_A11G2073
AP2 sgRNA2	D11	46804917	46804936	TTATACCATCGGACTTTGCTAGG	TCTTACCACCAGACTTTGCATGG	+	5	0.3142	Gh_D11G2379
AP2 sgRNA2	D01	56732921	56732940	TTATACCATCGGACTTTGCTAGG	TTATGGAATCGAACTTTGCTTGG	-	4	0.252	Gh_D01G1880
AP2 sgRNA2	A13	68637067	68637086	TTATACCATCGGACTTTGCTAGG	TTATATCTTTGGACTCTTCTGGG	+	5	0.1665	Gh_A13G1326
MYB44 sgRNA1	D03	2632420	2632439	TTGGCTGCTCACTGTGTTTATGG	TTGACTGCCCATTGTGTTTAAGG	+	3	0.4473	Intergenic
MYB44 sgRNA1	A07	39572231	39572250	TTGGCTGCTCACTGTGTTTATGG	TTAACTGTGCACTGTGTTTACGG	+	4	0.3656	Intergenic
MYB44 sgRNA1	A05	58468963	58468982	TTGGCTGCTCACTGTGTTTATGG	TTGGCTGCACAATTTATTTAGGG	+	4	0.1769	Intergenic
MYB44 sgRNA1	A09	44653886	44653905	TTGGCTGCTCACTGTGTTTATGG	TTCGCTGCTCAAAATGTTTACGG	+	4	0.1426	Intergenic
MYB44 sgRNA1	A07	10257230	10257249	TTGGCTGCTCACTGTGTTTATGG	TTGGCTAATCACGGTGTTTGGGG	+	4	0.1297	Intergenic
MYB44 sgRNA1	A02	10644862	10644881	TTGGCTGCTCACTGTGTTTATGG	TTTGCTATACACTGTGTTTATAG	+	4	0.1053	Gh_A02G0658
MYB44 sgRNA1	D09	10467714	10467733	TTGGCTGCTCACTGTGTTTATGG	ATAGATGTTCACTATGTTTACGG	-	5	0.2812	Gh_D09G0309
MYB44 sgRNA1	D06	1191999	1192018	TTGGCTGCTCACTGTGTTTATGG	TTGGCTGCTCACGGTGTTTATGG	-	1	0.2609	Gh_D06G0115
MYB44 sgRNA1	A02	74292866	74292885	TTGGCTGCTCACTGTGTTTATGG	ACGACTGCTATCTGTGTTTAGGG	+	5	0.2057	Gh_A02G1255
MYB44 sgRNA1	D13	57074404	57074423	TTGGCTGCTCACTGTGTTTATGG	TTGGATACTAAATGTGATTATGG	+	5	0.1887	Gh_D13G2148
MYB44 sgRNA2	A07	64828980	64828999	AACCCCCCACCACTCCCCGGTGG	TACCCCCAACCACTCCCTAGTGG	+	4	0.2985	intergenic
MYB44 sgRNA2	D10	919927	919946	AACCCCCCACCACTCCCCGGTGG	AACCCTCCCCCACTCCCTGTAGG	-	4	0.2388	intergenic
MYB44 sgRNA2	A12	719393	719412	AACCCCCCACCACTCCCCGGTGG	AACCCCCCACCCCTCCCAGAAGG	+	3	0.1328	intergenic
MYB44 sgRNA2	A05	9705620	9705639	AACCCCCCACCACTCCCCGGTGG	CCCCCCCCCCCACCCCCCGGTGG	+	4	0.11	intergenic
MYB44 sgRNA2	A09	7913489	7913508	AACCCCCCACCACTCCCCGGTGG	ATCCCCCCTCCACTCCTTGTCGG	-	5	0.0916	Gh_A09G0255
MYB44 sgRNA2	D09	7729127	7729146	AACCCCCCACCACTCCCCGGTGG	ATCCCCCCTCCACTCCTTGTCGG	-	5	0.0916	Gh_D09G0254
MYB44 sgRNA2	A02	5318938	5318957	AACCCCCCACCACTCCCCGGTGG	TAACCACCACCACTCCCTTGTAG	+	5	0.0894	Gh_A02G0411
MYB44 sgRNA2	D02	6100141	6100160	AACCCCCCACCACTCCCCGGTGG	TAACCACCACCACTCCCTTGTAG	+	5	0.0894	Gh_D02G0464
ARC sgRNA1	A06	35657190	35657209	GTCTACCATCAGTGTTCCCACGG	AGATACCATAAGTGTTCCCAAGG	-	4	0.5678	Intergenic
ARC sgRNA1	A05	52470896	52470915	GTCTACCATCAGTGTTCCCACGG	GGCTACCACTAGTGTTCTCATGG	-	4	0.4691	Intergenic
ARC sgRNA1	D02	8172071	8172090	GTCTACCATCAGTGTTCCCACGG	CTCTAACATCAGTGTCCCCATGG	-	3	0.4422	Intergenic
ARC sgRNA1	A09	64611259	64611278	GTCTACCATCAGTGTTCCCACGG	ATTTACCATTACTGTTCCCAAGG	-	4	0.3083	Gh_A09G1251
ARC sgRNA1	D09	39579827	39579846	GTCTACCATCAGTGTTCCCACGG	ATTTACCATTACTGTTCCCAAGG	-	4	0.3083	Gh_D09G1252
ARC sgRNA1	A01	4204724	4204743	GTCTACCATCAGTGTTCCCACGG	GTCAACCATCAGTGTTCCTACGG	+	2	0.2198	Gh_A01G0315
ARC sgRNA1	A01	4293938	4293957	GTCTACCATCAGTGTTCCCACGG	GTCAACCATCAGTGTTCCTACGG	+	2	0.2198	Gh_A01G0316
ARC sgRNA1	D05	51038057	51038076	GTCTACCATCAGTGTTCCCACGG	GTCCACAATTAGTGTTCCAACGG	+	4	0.1957	Gh_D05G3257
ARC sgRNA1	D01	54334257	54334276	GTCTACCATCAGTGTTCCCACGG	GTCGACCATCAATGTCCCAAAGG	+	4	0.1667	Gh_D01G1755
ARC sgRNA1	A09	69004726	69004745	GTCTACCATCAGTGTTCCCACGG	GTCTGCCAGAAATGTTCCAAGGG	+	5	0.1545	Gh_A09G1568
ARC sgRNA1	D09	43992591	43992610	GTCTACCATCAGTGTTCCCACGG	GTCTGCCAGAAATGTTCCAAGGG	+	5	0.1545	Gh_D09G1639
ARC sgRNA1	D05	53886017	53886036	GTCTACCATCAGTGTTCCCACGG	GTCCACAGTCAGTGTTCCAACGG	+	4	0.1525	Gh_D05G3336
ARC sgRNA2	D05	32956550	32956569	TCATAATCCTCGATGATTTGTGG	TCATAATTCTCGATGATCTATGG	+	3	0.3516	Intergenic
ARC sgRNA2	D05	32956550	32956569	TCATAATCCTCGATGATTTGTGG	TCATAATTCTCGATGATCTATGG	+	3	0.3516	Intergenic
ARC sgRNA2	D09	21741173	21741192	TCATAATCCTCGATGATTTGTGG	TCATAATCTCAGATGATTTGTGG	+	3	0.35	Intergenic
ARC sgRNA2	D06	712655	712674	TCATAATCCTCGATGATTTGTGG	TCATTATCCTCGATAATTTGTGG	+	2	0.3422	Intergenic
ARC sgRNA2	A10	92192631	92192650	TCATAATCCTCGATGATTTGTGG	ACATAGTAATCAATGATTTGGGG	+	5	0.3714	Gh_A10G1768
ARC sgRNA2	D10	56238563	56238582	TCATAATCCTCGATGATTTGTGG	ACATAGTAATCAATGATTTGGGG	+	5	0.3714	Gh_D10G2042
ARC sgRNA2	D12	36164050	36164069	TCATAATCCTCGATGATTTGTGG	TTATAATGCAAGATGATTTACGG	-	5	0.3522	Gh_D12G1035
ARC sgRNA2	D01	3884189	3884208	TCATAATCCTCGATGATTTGTGG	TCGTAATCCTCGATGATCTATGG	-	3	0.2455	Gh_D01G0350
ARC sgRNA2	A13	31832210	31832229	TCATAATCCTCGATGATTTGTGG	GCATAAGCATCAATGAATTGAGG	-	5	0.2401	Gh_A13G0759
ARC sgRNA2	A13	31833520	31833539	TCATAATCCTCGATGATTTGTGG	GCATAAGCATCAATGAATTGAGG	-	5	0.2401	Gh_A13G0759
ARC sgRNA2	D13	18053006	18053025	TCATAATCCTCGATGATTTGTGG	GCATAAGCATCAATGAATTGAGG	-	5	0.2401	Gh_D13G0892
ARC sgRNA2	A01	4240858	4240877	TCATAATCCTCGATGATTTGTGG	TCGTAATCCTCAATGATCTATGG	-	4	0.2292	Gh_A01G0316
ARC sgRNA2	D13	47057685	47057704	TCATAATCCTCGATGATTTGTGG	TCAAAATCCCCAATGATTTAGGG	+	4	0.2222	Gh_D13G1522
ARC sgRNA2	A07	39700638	39700657	TCATAATCCTCGATGATTTGTGG	ACTTTAACCTCGATAATTTGAGG	+	5	0.2114	Gh_A07G1425
ARC sgRNA2	A01	4203983	4204002	TCATAATCCTCGATGATTTGTGG	TCATAATCCTTGATGATATGTGG	-	2	0.2051	Gh_A01G0315
ARC sgRNA2	A01	4293200	4293219	TCATAATCCTCGATGATTTGTGG	TCATAATCCTTGATGATATGTGG	-	2	0.2051	Gh_A01G0316
ARC sgRNA2	A07	5177225	5177244	TCATAATCCTCGATGATTTGTGG	TCATAAAACTCAATGAATTTGGG	+	5	0.1982	Gh_A07G0407
ARC sgRNA2	D07	5050977	5050996	TCATAATCCTCGATGATTTGTGG	TCATAAAACTCAATGAATTTGGG	+	5	0.1982	Gh_D07G0472

Table S8 Summary of new off-targets and PAMs in WT plants.

New off-targets in WT	chromosome	start	end	Variation site	Ref	WT	DP (s79/s195/199)	off-target position
AP2 sgRNA1 NGG 20bp	A05	19502751	19502770	19502756	T	G	25/59/52	6
AP2 sgRNA1 NGG 20bp	A06	34541568	34541587	34541580	T	G	32/55/39	13
AP2 sgRNA1 NGG 20bp	A09	8396135	8396154	8396150	C	G	21/38/52	16
AP2 sgRNA1 NGG 20bp	D12	18543548	18543567	18543562	C	T	0/100/0	15
AP2 sgRNA1 NGA 20bp	A06	32225104	32225123	32225107	G	C	34/36/21	4
AP2 sgRNA1 NGA 20bp	A12	73319510	73319529	73319523	C	T	45/54/47	14
AP2 sgRNA2 NGG 20bp	A08	6786497	6786516	6786505	C	T	31/44/49	9
AP2 sgRNA2 NGG 20bp	A12	10740435	10740454	10740435	A	C	22/41/40	1
AP2 sgRNA2 NGG 20bp	A13	68637067	68637086	68637084	T	A	0/43/34	18
AP2 sgRNA2 NGG 20bp	scaffold206_A02	100667	100686	100679	T	C	16/13/10	13
AP2 sgRNA2 NGG 20bp	scaffold2075_A08	676457	676476	676457	T	G	39/44/39	1
AP2 sgRNA2 NGG 20bp	A07	45048982	45049001	45048989	T	TC	68/58/58	8
AP2 sgRNA2 NGG 20bp	A08	6731830	6731849	6731836	GTCGGA	G	20/36/35	7
AP2 sgRNA2 NGG 3bp (PAM)	D04	41357959	41357961	41357960	T	G	21/21/19	2
AP2 sgRNA2 NGG 3bp (PAM)	scaffold1170_A05	157085	157087	157086	G	C	27/24/16	2
AP2 sgRNA2 NGG 3bp (PAM)	A13	44505964	44505966	44505964	TG	T	40/27/18	1
AP2 sgRNA2 NAG 20bp	A02	74603143	74603162	74603155	A	C	21/40/56	13
AP2 sgRNA2 NAG 20bp	D10	56504343	56504362	56504354	C	T	36/48/33	12
AP2 sgRNA2 NGA 20bp	A01	80721063	80721082	80721072	C	T	26/23/23	10
AP2 sgRNA2 NGA 20bp	A10	24825259	24825278	24825262	T	G	38/42/42	4
MYB44 sgRNA1 NGG 20bp	A02	54133019	54133038	54133024	T	C	26/48/45	6
MYB44 sgRNA1 NGG 20bp	A08	43436791	43436810	43436806	G	C	23/25/27	16
MYB44 sgRNA1 NGG 20bp	A08	58915777	58915796	58915792	G	C	16/0/11	16
MYB44 sgRNA1 NGG 20bp	A10	60143064	60143083	60143077	G	A	23/33/25	14
MYB44 sgRNA1 NGG 20bp	A10	60143064	60143083	60143079	G	C	24/33/25	16
MYB44 sgRNA1 NGG 20bp	A12	28745286	28745305	28745297	C	A	23/17/22	12
MYB44 sgRNA1 NGG 20bp	D04	41447227	41447246	41447229	G	T	23/50/40	3
MYB44 sgRNA1 NGG 20bp	scaffold1168_A05	481901	481920	481903	C	T	2/0/0	3
MYB44 sgRNA1 NGG 20bp	scaffold1176_A05	1060638	1060657	1060649	T	C	27/30/40	12
MYB44 sgRNA1 NGG 20bp	D01	54158410	54158429	54158414	CACAATT	C	2029/9/7	5
MYB44 sgRNA1 NGG 20bp	D03	902611	902630	902622	CT	C	37/80/50	12
MYB44 sgRNA1 NGG 20bp	scaffold3436_A13	13844	13863	13854	A	AAT	33/22/25	11
MYB44 sgRNA1 NGG 3bp (PAM)	A13	59873391	59873393	59873391	G	A	31/39/26	1
MYB44 sgRNA1 NGA 20bp	A02	80229603	80229622	80229604	T	G	38/42/27	2
MYB44 sgRNA1 NGA 20bp	A08	30355690	30355709	30355703	G	T	23/31/19	14
MYB44 sgRNA1 NGA 20bp	D09	22090127	22090146	22090131	C	T	27/56/34	5
MYB44 sgRNA2 NGG 20bp	A09	7913489	7913508	7913504	G	T	28/41/43	16
MYB44 sgRNA2 NGG 20bp	A13	9292818	9292837	9292826	A	AG	0/12/0	9
MYB44 sgRNA2 NGG 20bp	D06	1191807	1191826	1191809	A	AC	32/51/44	3
MYB44 sgRNA2 NGG 20bp	D10	919927	919946	919935	T	TA	14/34/30	9
MYB44 sgRNA2 NAG 20bp	D04	50384789	50384808	50384797	ACC	A	27/32/40	9
ARC sgRNA1 NGG 20bp	A07	16120505	16120524	16120509	A	C	24/50/37	5
ARC sgRNA1 NGG 20bp	A08	46070347	46070366	46070350	A	G	36/54/44	4
ARC sgRNA1 NGG 20bp	A08	53472735	53472754	53472753	C	T	29/27/20	19
ARC sgRNA1 NGG 20bp	A13	50998870	50998889	50998887	G	A	57/51/0	18
ARC sgRNA1 NGA 3bp (PAM)	scaffold757_A04	92495	92497	92496	A	G	20/23/28	2
ARC sgRNA2 NGG 20bp	A08	26503749	26503768	26503758	G	A	24/35/19	10
ARC sgRNA2 NGG 20bp	A08	51413373	51413392	51413383	A	G	29/35/33	11
ARC sgRNA2 NGG 20bp	A08	70517445	70517464	70517445	T	C	26/22/29	1
ARC sgRNA2 NGG 20bp	A09	21582506	21582525	21582520	G	T	22/36/17	15
ARC sgRNA2 NGG 20bp	D02	19915669	19915688	19915670	T	C	20/36/39	2
ARC sgRNA2 NGG 20bp	D05	55270261	55270280	55270261	T	C	0/33/27	1
ARC sgRNA2 NGG 20bp	D05	55577854	55577873	55577867	T	C	24/57/34	14
ARC sgRNA2 NGG 20bp	D06	10843828	10843847	10843828	C	T	33/56/52	1
ARC sgRNA2 NGG 3bp (PAM)	D05	54534661	54534663	54534662	A	G	0/51/45	2
ARC sgRNA2 NAG 20bp	A08	54198548	54198567	54198561	A	C	24/7/9	14
ARC sgRNA2 NAG 20bp	A08	58582960	58582979	58582977	T	C	34/50/28	18
ARC sgRNA2 NAG 20bp	A10	21886201	21886220	21886216	T	C	19/29/32	16
ARC sgRNA2 NGA 20bp	A01	53924175	53924194	53924185	C	T	20/22/33	11
ARC sgRNA2 NGA 20bp	A06	32800431	32800450	32800431	C	G	0/45/52	1
ARC sgRNA2 NGA 20bp	scaffold3629_A13	17619	17638	17632	T	C	25/50/28	14

The production genetic variations in wild-type (WT) can creat and break new off-target sites and PAMs. These potential off-target site involved NGG, NAG, and NGA of PAM types. The 'Ref', 'WT', 'DP', 'off-target position' represent the reference genome site, WT genome genetic variation, sequencing depth, and off-target position relative reference genome position.

Table S9 Primers used for on-targets and off-targets.

Loci	Target	Primers
-	Cas9	F: 5' AACCTACAACTGTGGTAAATCA 3'
		R: 5' GTGATCCAAATTGAGACTTAGG 3'
Gh_D13G2207	AP2	F: 5' GCAGTACAGATGGAACCAGGAG 3'
	sgRNA1/2	R: 5' CCCGGATAGATTTGCACTTT 3'
Gh_A06G0606	MYB44	F: 5' CATCGGAGTCATCAAGGATCAAAG 3'
	sgRNA1/2	R: 5' TAGCAATCACACCCTTCATCGC 3'
Gh_A06G0136	ARC	F: 5' ATGCCCGAGTCTTTCTTTATGG 3'
	sgRNA1/2	R: 5' ACAGCCCTTCCCATTTTCAAC 3'
D01: 55912563-55913203	OFTM1	F: 5' ATAAGCTGACGTGGCACGCT 3'
		R: 5' GGGCTGTATGGCTAAGGATTTG 3'
D06:1191487-1192127	OFTM2	F: 5' TAAGGACCTATCAAGCAAATTCACC 3'
		R: 5' TTTACAAAAGCGGAGGATGAGAC 3'
A01:4321712-4322352	OFTM3	F: 5' TTCAAGGCGTTTTCTACATTTGTC 3'
		R: 5' TTTCATTTCTTTTTGTGGTTCCC 3'
A01:4381686-4382326	OFTM4	F: 5' AGTAGGACACAAACCAAAGCATCT 3'
		R: 5' ATGAGGATATTTTCATCACTTCATAAA 3'
